# Screening for hypertension in a public dental clinic: A single-centre cross-sectional study in Australia

**DOI:** 10.1371/journal.pone.0351629

**Published:** 2026-06-11

**Authors:** Shalinie King, Simone Marschner, Haeri Min, Alice Gibson, Niamh Chapman, Clara K. Chow

**Affiliations:** 1 The Sydney Dental School, Faculty of Medicine and Health, Faculty of Medicine and Health, The University of Sydney, Sydney, Australia; 2 Westmead Applied Research Centre, Faculty of Medicine and Health, The University of Sydney, Sydney, Australia; 3 The Charles Perkins Centre, The University of Sydney, Sydney, Australia; 4 Leeder Centre for Health Policy, Economics and Data, School of Public Health, Faculty of Medicine and Health, University of Sydney, Sydney, Australia; 5 Sydney School of Health Sciences, Faculty of Medicine and Health, The University of Sydney, Sydney, Australia; 6 The Menzies Institute for Medical Research, College of Health and Medicine, University of Tasmania, Hobart, Australia; 7 The Sydney Medical School, Faculty of Medicine and Health, The University of Sydney, Sydney, Australia; Ehime University Graduate School of Medicine, JAPAN

## Abstract

**Background:**

Hypertension affects over 1.3 million people globally and markers of poor oral health have been associated with hypertension.

**Objective:**

This study aimed to determine hypertension awareness, treatment and control, among adults attending a public dental clinic, and explore age-related differences between younger (< 65 years) and older adults (≥ 65 years) including associations between markers of oral health and hypertension.

**Method:**

This cross-sectional study recruited adults ≥18 years from an Australian public dental clinic between November 2022 and May 2023. Two blood pressure readings were obtained using an automated device and the average used for analyses. Demographic, oral health and medical history details were obtained via survey. Hypertension was defined as a systolic blood pressure (SBP) ≥140 mmHg and/or diastolic blood pressure (DBP) ≥90 mmHg, or being aware (self-reported diagnosis), and treated with anti-hypertensive medication.

**Results:**

Participants (n = 302) were middle aged (mean 59.9 ± 17.5 years, 51.5% ≥ 65 years, 60.7% female), one third had significant tooth loss (<20 teeth), half required treatment for periodontitis and 52.0% had hypertension (95% CI: 46.2%, 57.7%). Of those with hypertension (n = 157), 82.7% were aware, 76.9% were treated for hypertension, and 56.6% of those treated had controlled blood pressure. Although older adults were more likely than younger adults to have hypertension (74.2% vs 28.1%), younger adults were less likely to be aware and treated (70.7% vs 86.8%, 56.1% vs 86.8%, respectively). Markers of poor oral health were not independently associated with hypertension.

**Conclusions:**

In this population with a high burden of oral disease, hypertension was common highlighting the importance of opportunistic blood pressure assessment. Dental settings may offer an additional point of contact for identifying individuals with undiagnosed or uncontrolled hypertension, particularly among younger adults with lower awareness.

## Introduction

Hypertension is the leading modifiable risk factor for cardiovascular disease (CVD). It is often referred to as a ‘silent killer’ as it typically presents with minimal symptoms yet causes significant damage to major organs such as the heart and kidneys, leading to premature death [1]. Globally, high blood pressure affects over 1.3 billion people worldwide and is responsible for approximately 10 million deaths annually contributing to nearly 20% of all deaths globally [2]. Globally, an estimated 46% of adults with hypertension are unaware of their diagnosis and, even amongst those receiving treatment for hypertension, only 21% have well-controlled blood pressure [2]. In Australia, approximately one in three adults (22–34%) have hypertension [3,4], and of those 32% have well controlled blood pressure [4]. Nationally, hypertension contributes to over 25,000 deaths annually as a result of heart disease, stroke, heart failure, kidney disease and dementia [2].

Early detection and effective management of hypertension can reduce mortality and morbidity, hence the importance of screening programs and regular health checks [5]. Screening dental patients presents an opportunity to detect previously undiagnosed or uncontrolled hypertension, offering a potential avenue for integrating oral health services into primary care [6,7]. The majority of dentists and dental hygienists do not routinely monitor blood pressure [8] therefore in 2006 the American Dental Association reaffirmed its 1974 recommendation that dental practitioners routinely measure blood pressure for all new patients and annually on follow-up visits to improve the early detection and management of hypertension [9].

Oral diseases such as periodontitis are local inflammatory conditions that trigger chronic systemic inflammation and elevated risk of hypertension [10]. Previous studies have shown markers of oral health, including tooth loss, poor oral hygiene, and periodontitis to be associated with hypertension in people under the age of 65 years [11]. These findings support the need for integrated care delivery.

Public dental services in Australia, provide dental care to those eligible for health benefits often with additional social determinants that may signify a higher cardiovascular risk. This study examined the clustering of oral health and hypertension in a large public dental service serving a diverse and socioeconomically disadvantaged population in Sydney, Australia.

The aim of this study was to determine hypertension awareness, treatment and control, among adults attending a public dental service, and explore age‑specific patterns and associations with oral health. We hypothesised that there would be a high proportion of patients with hypertension, and that markers of poor oral health would be associated with hypertension.

## Methods

Participants in this cross-sectional study were recruited from a public dental assessment service conducted at the Westmead Centre for Oral Health, Western Sydney, Australia. The centre provides care for people on low incomes seeing on average about 50,000 patients/year. The study was approved by the Western Sydney Local Health District Human Ethics Committee (protocol no: 2023/ETH01673). Informed consent was obtained from all participants prior to data collection. The study conforms to the STROBE guidelines.

### Study population and setting

Consecutive participants attending the clinic were approached by clinic staff on 3 of 5 days per week between 21^st^ November 2023 and 23^rd^ April 2024. The inclusion criteria were adults aged ≥18 years attending the dental clinic and, able to read/understand English. The exclusion criteria were cognitive impairment that affected the ability to understand instructions and not read/understand English. The purpose of the assessment service is to assess patients’ treatment needs so that they can be triaged and coded for the dental waiting list. The visit involved a basic dental examination and did not include the provision of any dental treatment. Once the clinical assessment was completed, the treating clinician referred patients who were interested in participating in the study to a member of the study team (SK) who explained the study process and completed the electronic consent form [12] prior to data collection.

### Data collection

A survey administered by research study staff was used to obtain demographic characteristics (age, ethnicity, and language other than English), medical history, oral health information and cardiovascular disease risk related factors. The survey was delivered through the research electronic data capture (REDCap) tools hosted at the Western Sydney Local Health District [13,14]. BP was measured in a dental chair and physical measurements (height and weight) were conducted in the dental clinical room. The process of data collection took approximately 10–15 minutes per participant.

### Oral health

Oral health information collected included risk factors for dental decay (oral hygiene, fluoride exposure, sugar intake, presence of dry mouth and frequency of snacking) [15], risk factors for periodontitis [oral hygiene, smoking, harmful alcohol intake, type 2 diabetes mellitus (T2DM) and obesity] [16], oral health treatment needs (number of fillings required and the need for periodontal treatment) and a tooth count. Oral health-related treatment needs and the number of teeth as indicated by the clinician, were collected from the clinical records. Periodontal treatment needs were determined using the periodontal screening and recording index (PSR), which provides a score for the periodontal treatment needs [17]. Briefly, a score of 0 indicated no treatment needs, scores of 1–2 indicate simple treatment needs for gingivitis, scores of 3–4 indicate the need for treatment of periodontitis. All other data were self-reported.

### Cardiovascular health

Cardiovascular risk information was collected via a survey and included previous diagnosis of elevated cholesterol levels, hypertension, T2DM, prescription of lipid-lowering and blood pressure-lowering medications, history of atrial fibrillation, fruit and vegetable intake and exercise and was based on recommended CVD risk assessment guidelines [18,19].

Physical measurements including blood pressure, height and weight enabling calculation of body mass index (BMI) (kg/m^2^) were also performed. To measure height and weight, the participants were asked to remove their shoes and any bulky clothing. Height was measured using a stadiometer, and weight was measured using electronic scales.

### Blood pressure measurement

Blood pressure was measured using a validated automated device (Omron HEM-907®) with the participant seated upright in the dental chair after resting for 5 minutes with their arm supported at heart level using a moveable table. The BP measurements represent real-world clinical measurements and are not standardised research-grade measurements recommended by hypertension guidelines [20].Two consecutive BP measurements were made on the right arm unless contraindicated (e.g., injury) using the appropriate cuff size as determined by the reference range of the cuff (2 cuff sizes were available, 22–32 cm and 32–42 cm).

### Definitions

Hypertension for this study was defined as either 1) systolic blood pressure (SBP) ≥140 mmHg and/or diastolic blood pressure (DBP) ≥90 mmHg, or 2) aware and treated with anti-hypertensive medication. Hypertension awareness was defined as a self-reported diagnosis of hypertension. Hypertension treatment was defined by self-reported anti-hypertensive therapy. Hypertension control was defined as a SBP < 140 mmHg and DBP < 90 mmHg and reported as a percentage of all patients with hypertension and as a percentage of those taking anti-hypertensive medication. Overweight was defined as BMI ≥ 25 kg/m^2^ and obesity as BMI ≥ 30 kg/m^2^.

### Statistical analysis

Continuous variables are presented as mean ± SD and categorical variables as numbers and percentages. The proportion of patients with hypertension (primary outcome) was presented as a proportion of the total sample with associated 95% confidence intervals (CI). Differences in proportions between groups were calculated using a chi-square test. Logistic regression was used to identify oral health characteristics associated with hypertension (secondary outcome). All data obtained from the survey were used in the analysis and the level of missingness was reported for each covariate. The models were adjusted for age, sex, smoking status and T2DM as prespecified. Statistical significance was set at *P* < 0.05, Analyses were performed using R software (version 4.4.0).

### Sample size calculation

For the expected proportion of high measured BP of 22.8% which represented the national prevalence of high measured BP in Australia [21], the required sample size is 302 for the margin of error or absolute precision of ± 5% in estimating the prevalence with 95% confidence and considering the potential loss/attrition of 10%. With this sample size the anticipated 95% confidence interval is (17.8%, 27.8%). The sample size was calculated using scalex SP calculator [22].

## Results

**Fig 1:**
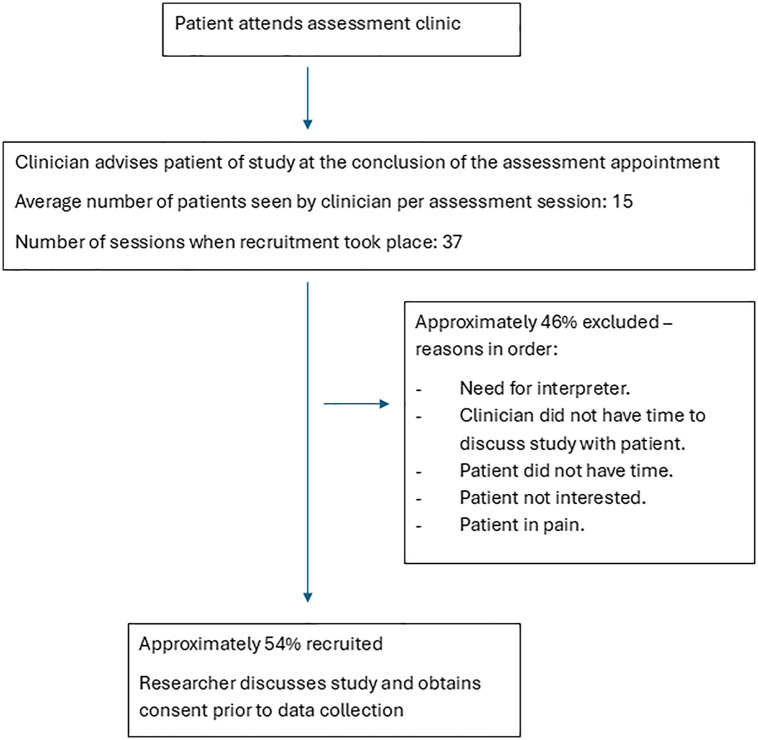
Study flow chart. Participant recruitment, inclusion and exclusion and final sample included in the study.

Over the study period, approximately 54% of patients attending the clinic were recruited to the study (n = 302). One of the main reasons for exclusion was the need for an interpreter, other reasons included lack of participant time or interest in participation or if the individual was in pain (Fig 1).

Participants were middle aged (mean 59.9 ± 17.5 years), 60.7% were female and 72.2% spoke a language in addition to English. The majority (84.4%) had a low income of between $300–999 per week, and almost one third had a level of educational attainment of high school or lower. The majority (>80.0%) had seen their medical practitioner within the last 12 months for blood pressure, cholesterol and blood glucose measurement (Table 1).

**Table 1:** Characteristics of study population.

Characteristic	Total	<65	≥ 65	p-value
	N = 302	N = 146 (48.5%)	N = 155 (51.5%)	
**Demographic characteristics**				
**Age** Missing = 1	59.9 (17.5)	44.9 (12.5)	74.1 (6.0)	**<0.001**
**Sex** Missing = 4				0.09
Male	117 / 298 (39.3%)	49 / 144 (34.0%)	67 / 153 (43.8%)	
Female	181 / 298 (60.7%)	95 / 144 (66.0%)	86 / 153 (56.2%)	
**Speak a language other than English**	218 / 302 (72.2%)	99/146 (67.8%)	118/155 (76.1%)	1.08
**Aboriginal or Torres Strait Islander origin** Missing = 5	8 / 297 (2.7%)	7/142 (4.9%)	1/154 (0.6%)	**0.03**
**Income** Missing = 1				**<0.001**
Dont know	3 / 301 (1.0%)	0/146 (0.0%)	3/154 (3.9%)	
<$300/w ($16,000/y)	32 / 301 (10.6%)	26/146 (17.8%)	6/154 (3.9%)	
$300-$999/w ($16,000-$51,999/y)	254 / 301 (84.4%)	111/146 (76.0%)	142/154 (92.2%)	
≥ $1000 (≥ $52,000/y)	8 / 301 (2.7%)	7/146 (4.8%)	1/154 (0.6%)	
Prefer not to say	4 / 301 (1.3%)	2/146 (1.4%)	2/154 (1.3%)	
**Highest level of education** Missing = 1				**0.004**
Primary School	35 / 301 (11.6%)	8/146 (5.5%)	27/154 (17.5%)	
Secondary School	148 / 301 (49.2%)	73/146 (23.3%)	75/154 (48.7%)	
Certificate/diploma/TAFE	56 / 301 (18.6%)	34/146 (23.3%)	21/154 (20.1%)	
University	62 / 301 (20.6%)	31/146 (21.2%)	31/154 (20.1%)	
**Health Service Utilisation**				
**Last medical check up**				**<0.001**
≥ 1 year ago	44 / 302 (14.6%)	34/146 (23.2%)	10/155 (6.5%)	
Less than 12 months ago	258 / 302 (85.4%)	112 / 146 (76.7%)	145 / 155 (93.5%)	
**Last blood pressure check**				**<0.001**
≥ 1 year ago	42 / 302 (13.9%)	34/146 (13.3%)	8/155 (5.1%)	
Less than 12 months ago	260 / 302 (86.1%)	112 / 146 (76.7%)	147 / 155 (94.8%)	
**Last cholesterol check**				**<0.001**
≥ 1 year ago	54 / 302 (17.9%)	40/146 (27.4%)	14/155 (9.0%)	
Less than 12 months ago	248 / 302 (82.1%)	106 / 146 (72.6%)	141 / 155 (91.0%)	
**Oral Health**				
**Tooth brushing frequency** Missing = 1				**0.04**
Less than once a day	22 / 301 (7.3%)	16 / 145 (11.0%)	6 / 155 (3.9%)	
Once a day	136 / 301 (45.2%)	67 / 145 (46.2%)	69 / 155 (44.5%)	
Twice or more times per day	143 / 301 (47.5%)	62 / 145 (42.8%)	80 / 155 (51.6%)	
**Use floss or use interdental brushes** Missing = 6	167 / 296 (56.4%)	82 / 144 (56.9%)	84 / 151 (55.6%)	0.820
**Drink fluoridated tap water** Missing = 1	248 / 301 (82.4%)	114 / 145 (78.6%)	133 / 155 (85.8%)	0.103
**Use a standard fluoride containing toothpaste at least once a day** Missing = 6	281 / 296 (94.9%)	136 / 145 (93.8%)	144 / 150 (96.0%)	0.34
**Drink at least 2L of water every day**	131 / 302 (43.4%)	67 / 146 (45.9%)	63 / 155 (40.6%)	0.36
**Add sugar to tea/coffee** Missing = 3	154 / 299 (51.5%)	77 / 145 (53.1%)	76 / 153 (49.7%)	0.56
**Sugar sweetened beverages** Missing = 1				**0.009**
Twice or more times per day,	39 / 301 (13.0%)	26 / 146 (17.8%)	13 / 154 (8.4%)	
Once a day	33 / 301 (11.0%)	19 / 146 (13.0%)	14 / 154 (9.1%)	
I don’t drink sugar sweetened beverages	95 / 301 (31.6%)	35 / 146 (24.0%)	60 / 154 (39.0%)	
Less than once a day	134 / 301 (44.5%)	66 / 146 (45.2%)	67 / 154 (43.5%)	
**Snack frequently** **(more than 3 times a day)** Missing = 2	83 / 300 (27.7%)	52 / 146 (35.6%)	31 / 153 (20.3%)	**0.003**
**Medications that make your mouth dry** Missing = 5	124 / 297 (41.8%)	55 / 142 (38.7%)	68 / 154 (44.2%)	0.34
**Number of teeth present (clinician reported)**				**<0.001**
Number of teeth (mean)	21.2 (8.9)	24.9 (7.0)	17.7 (9.0)	
< 20 teeth	100 /302 (33.1%)	24/146 (16.4%)	76/155 (49.1%)	
≥ 20 teeth	202 / 302 (66.9%)	122 / 146 (83.6%)	79 / 155 (51.0%)	
**Number of fillings needed (clinician reported)** Missing = 8	1.8 (2.5)	2.0 (2.5)	1.6 (2.4)	0.15
**PSR code (clinician reported)** Missing = 15				0.635
0-2	145 / 287 (50.5%)	73 / 141 (51.8%)	71 / 145 (49.0%)	
3-4	142 / 287 (49.5%)	68 / 141 (48.2%)	74 / 145 (51.0%)	
**Cardiovascular Health**				
**BMI (mean)** Missing = 2	29.2 (6.3)	29.6 (6.8)	28.9 (5.7)	0.311
Underweight	3 / 300 (1.0%)	3 / 145 (2.1%)	0 / 154 (0.0%)	
Healthy weight	73 / 300 (24.3%)	31 / 145 (21.4%)	41 / 154 (26.6%)	
Overweight	106 / 300 (35.3%)	50 / 145 (34.5%)	56 / 154 (36.4%)	
Obese	106 / 300 (35.3%)	61 / 145 (42.1%)	57 / 154 (37.0%)	
**Systolic blood pressure (mm/hg)** Missing = 2	127.8 (19.5)	120.5 (17.5)	134.8 (18.8)	**<0.001**
**Diastolic blood pressure (mm/hg)** Missing = 2	74.5 (11.4)	76.3 (10.6)	72.8 (11.9)	**0.007**
**High cholesterol** Missing = 3	140 / 299 (46.8%)	41 / 144 (28.5%)	98 / 154 (63.6%)	**<0.001**
**Medication to lower your cholesterol** Missing = 163	116 / 139 (83.5%)	27 / 41 (65.9%)	88 / 97 (90.7%)	**<0.001**
**Stroke or transient ischaemic attack (TIA)** Missing = 5	10 / 297 (3.4%)	2 / 142 (1.4%)	8 / 154 (5.2%)	0.106
**Heart attack** Missing = 1	12 / 301 (4.0%)	1/142 (0.7%)	11/155 (7.1%)	**0.006**
**Type 2 Diabetes Mellitus** Missing = 13	61 / 289 (21.1%)	15 / 140 (10.7%)	46 / 148 (31.1%)	**<0.001**
**Atrial fibrillation**	15 / 302 (5.0%)	5 / 146 (3.4%)	10 / 155 (6.5%)	0.228
**Smoking status** Missing = 1				**<0.001**
Never smoked	164 / 301 (54.5%)	75 / 146 (51.4%)	89 / 154 (57.8%)	
Previously smoked (stopped more than 1 year ago)	77 / 301 (25.6%)	24 / 146 (16.4%)	52 / 154 (33.8%)	
Currently smoke (or stopped less than or equal to 1 year ago)	60 / 301 (19.9%)	47 / 146 (32.2%)	13 / 154 (8.4%)	
**Lifestyle Factors**				
**Alcohol** Missing = 3				0.06
Never	173 / 299 (57.9%)	87 / 145 (60.0%)	85 / 153 (55.6%)	
Less than or equal to 2 standard drinks a day	116 / 299 (38.8%)	50 / 145 (34.5%)	66 / 153 (43.1%)	
More than 2 standard drinks a day	10 / 299 (3.3%)	8 / 145 (5.5%)	2 / 153 (1.3%)	
**Servings of vegetables**				**0.007**
None	13 / 302 (4.3%)	6 / 146 (4.1%)	7 / 155 (4.5%)	
1-4 serves daily	205 / 302 (67.9%)	87 / 146 (59.6%)	118 / 155 (76.1%)	
5 or more serves of vegetables per day	30 / 302 (9.9%)	16 / 146 (11.0%)	13 / 155 (8.4%)	
I dont eat vegetables everyday	54 / 302 (17.9%)	37 / 146 (25.3%)	17 / 155 (11.0%)	
**Servings of fruit**				**<0.001**
None	91 / 302 (30.1%)	58 / 146 (39.7%)	33 / 155 (21.3%)	
1 serve a day	86 / 302 (28.5%)	44 / 146 (30.1%)	42 / 155 (27.1%)	
2 or more serves a day	125 / 302 (41.4%)	44 / 146 (30.1%)	80 / 155 (51.6%)	
**Exercise** Missing = 8				0.789
Not at all	148 / 294 (50.3%)	70 / 141 (49.6%)	78 / 152 (51.3%)	
Less than 30 minutes 5 days a week	36 / 294 (12.2%)	16 / 141 (11.3%)	20 / 152 (13.2%)	
30 minutes or more 5 days a week	110 / 294 (37.4%)	55 / 141 (39.0%)	54 / 152 (35.5%)	

Mean (SD); n/N (%).

In terms of the risk factors for dental decay, just under half of the population (47.5%) brushed twice daily. Although the majority (82.4%) drank fluoridated water around half the population (43.4%) did not drink the recommended 2L of water per day, and nearly a quarter (24.0%) consumed sugar sweetened beverages (SSBs) once or more times per day. Regarding the risk factors specific for periodontitis, nearly three quarters of the cohort (74.2%) were overweight or obese with a mean BMI of 29.2 kg/m^2^, 21.1% reported a diagnosis of type 2 diabetes mellitus (T2DM) and 19.9% were current smokers. In terms of oral disease burden, 33.1% had significant tooth loss (< 20 teeth) and nearly half of the population (49.5%) required treatment for periodontal disease.

In relation to cardiovascular health, the mean SBP was 127.8 mmHg ± 19.5, and the mean DBP was 74.5 mmHg ± 11.4. A previous diagnosis of elevated cholesterol levels (self-reported diagnosis) was reported by 46.8%, of whom 83.5% were taking lipid lowering medications. A total of 3.4% reported a history of stroke, 4.0% reported a history of heart attack and 5.0% reported a diagnosis of atrial fibrillation.

Compared with older adults (≥ 65 years), younger adults (<65 years) were more likely to engage in poor overall health behaviours including smoking (32.2% vs 8.4%), harmful alcohol consumption (5.5% vs 1.3%), not consuming vegetables daily (25.3% vs 11.0%), and not eating fruit (39.7% vs 21.3%). They were also more likely to display poor oral health behaviours such as brushing once a day or less (57.2% vs 48.4%) (Table 1). Notably, the 35–44 year age group had the highest rates of poor health behaviours (S1 Table).

### Proportion with hypertension, and awareness, treatment and control of hypertension

The proportion of patients determined as having hypertension using our definition of either high measured blood pressure or self-reported history of hypertension and treatment, was 52.0% (95% CI: 46.2%, 57.7%). A greater proportion of older adults had hypertension than younger adults [74.2% (CI: 43.1%, 62.0%) vs 28.1% (21.1%, 36.2%)]. However, amongst those with hypertension, younger adults were more likely to record an elevated blood pressure reading at the time of measurement [70% (CI: 53.3%, 82.9%) vs 52.6% (CI: 43.1%, 62.0%)], Table 2.

**Table 2:** Proportion with hypertension, and awareness, treatment and control of hypertension.

Diagnostic categories of blood pressure	Total (n = 302)	<65 (n = 146)	≥ 65 (n = 155)	p-value
	n/N (%) 95%CI	n/N (%) 95%CI	n/N (%) 95%CI	
Determined hypertension* **[Number (%) with high measured blood pressure or aware and treated]**	157/302 (52.0%) (CI:46.2%, 57.7%)	41 / 146 (28.1%) (CI:21.1%, 36.2%)	115 / 155 (74.2%) (CI: 66.4%, 80.7%	**<0.0001**
**High measured blood pressure**				
[Number (%) of patients with a SBP ≥140 mmHg and DBP ≥90 mmHg among those with hypertension, missing= 2]	88 / 155 (56.8%) (CI: 48.6%, 64.6%)	28 / 40 (70.0%) (53.3%, 82.9%)	60 / 114 (52.6%) (43.1%, 62.0%)	0.06
**Hypertension awareness**				
[Number (%) of patients aware among those with hypertension, missing=1]	129/156 (82.7%) (CI:75.6%, 88.1%)	29 / 41 (70.7%) (CI:54.3%, 83.4%)	99 / 114 (86.8%) (CI:78.9%, 92.2%)	**0.02**
**Hypertension** **(Awareness and treatment)**				
[Number (%) of patients aware and treated among those with hypertension, missing = 1]	120/156 (76.9%) (CI: 69.4%, 83.1%)	23/41 (56.1%) (CI: 39.9%, 71.2%)	96/114 (84.2%) (CI: 75.9%, 90.1%)	**0.0003**
**Hypertension control**				
Number (%) with BP < 140/90 among overall population with hypertension (missing = 2)	67/ 155 (43.2%) (CI: 35.4%, 51.4%)	12 / 40 (30.0%) (CI:17.1%, 46.7%)	54 / 114 (47.4%) (CI: 38.0%, 56.9%)	0.06
Number (%) with BP < 140/90 among those taking anti-hypertensive medication (missing = 2)	67/ 118 (56.8%) (CI: 47.3%, 65.8%)	12 / 22 (54.5%) (CI:32.7%, 74.9%)	54 / 95 (56.8%) (CI:46.3%, 66.8%)	0.845

*Determined Hypertension - systolic blood pressure (SBP) ≥140 mmHg and/or diastolic blood pressure (DBP) ≥90 mmHg, based on the mean of 2 consecutive blood pressure readings or aware and treated with anti-hypertensive medication.

Of those with determined hypertension (n = 157), 82.7% were aware of their diagnosis, 76.9% were receiving antihypertensive medication (treated), and 56.5% of those on medication had their blood pressure controlled. Hypertension awareness, and treatment, were lower in younger compared to older adults. Table 2 displays the diagnostic categories of blood pressure for the overall population and age group.

### Association between oral health characteristics and hypertension

**Fig 2:**
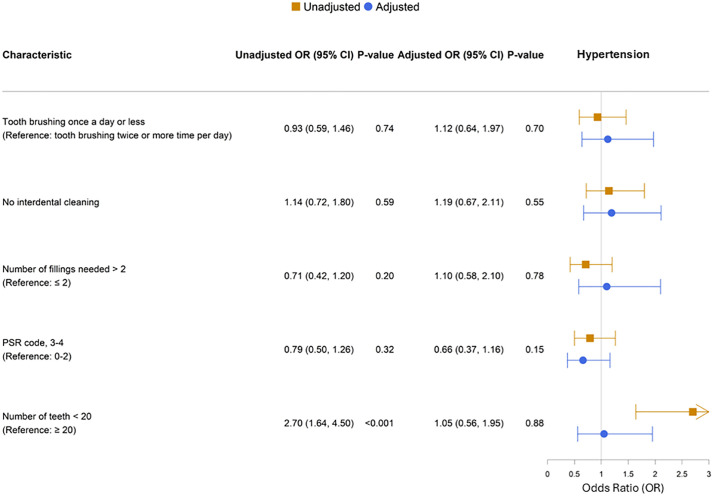
Association between markers of oral health and hypertension. Forest plot of unadjusted and adjusted odds ratios (OR) and 95% confidence intervals (CI) for the association between oral health characteristics and hypertension. Those with fewer than 20 teeth had greater odds than those with ≥ 20 teeth of having hypertension however, after adjusting for age, sex, smoking status and diabetes there was no evidence of association. [Periodontal Screening and Recording (PSR)].

Markers of oral hygiene including tooth brushing frequency and interdental cleaning were not associated with hypertension in the unadjusted (p = 0.80, p = 0.59) or adjusted models (p = 0.66, p = 0.55, respectively). Those with fewer than 20 teeth (n = 100) had greater odds than those with ≥ 20 teeth (n = 202) of having hypertension [2.70 OR; 95% CI,1.64,4.50] however, after adjusting for age, sex, smoking status and T2DM there was no evidence of association [1.05 OR; 95%CI,0.56,1.59]. There was also no association between periodontal treatment needs and hypertension in either the unadjusted [0.79 OR; 95%CI, 0.50, 1.26] or adjusted model [0.66 OR;95% CI, 0.37, 1.16] (Fig 2).

## Discussion

This cross-sectional study conducted in a public dental clinic in Western Sydney found a large burden of hypertension, with over one in two adults affected. A higher proportion of older (74.1%) compared to younger adults (28.1%) in this population had hypertension and while overall rates of awareness and treatment were high, younger adults were less likely than older adults to be aware and treated for hypertension (56.1% vs 84.2%, 30.0% vs 47.4%, respectively). These findings suggest that within public dental services there are opportunities to address broader cardiovascular disease prevention goals, especially among younger adults. This population also had a high burden of oral disease including significant tooth loss, a high need for periodontal treatment and a reduced frequency of preventive behaviours such as tooth brushing. The exploratory analysis did not identify a significant association between markers of poor oral hygiene, significant tooth loss or periodontal treatment needs and hypertension.

The proportion with hypertension in this study (52.0%) exceeded both the national average of 34% [4] and a previous Australian screening campaign (31.3%) [23], likely reflecting the older mean age of our sample (59.9 years vs 43.5 years). Significantly, our population had a high burden of oral disease with 33.1% reporting fewer than 20 teeth, higher than the national average of 22.2% for a 55–74 year age group and well above the overall average of 10.1% for those aged 15 years and above [24]. Furthermore, nearly one in two (49.5%) patients were identified as requiring treatment for periodontitis, compared to 30% in the national adult population [24]. They also had an increased risk of tooth decay, periodontitis and CVD with only 47.5%, brushing twice daily or more, below the national average of 51% [25]; high sugar intake with 24% consuming SSBs once or more times/day compared to the national average of 9.1% [26]; and high levels of T2DM, overweight or obesity, (21.1%, 74.2% respectively) compared to the corresponding national averages for each (5.1%, 66% respectively) [27–29]. Over 70% of the population spoke a language in addition to English, and the majority (80%) had a low income. This cohort’s higher hypertension rate likely reflects their greater oral and metabolic disease burden and the social determinants of health affecting this population.

The rates of awareness and treatment (82.7% and 76.9% respectively) reported in this study are higher than those reported in Australia (50.5% and 40.6% respectively) [23] or globally (46.5%, 40.6% respectively) [30] and likely reflect the older population in this study. Older adults have been found to have better awareness and higher treatment rates compared to younger adults [30]. Similarly, despite the small sample size, we found that younger individuals were more likely than older individuals to be unaware of their condition, and untreated (29.3% vs 13.2%, 43.9% vs 15.8%, respectively). Hence, screening patients in Australian public dental clinics, especially younger adults is an opportunity to identify and refer those with undiagnosed or uncontrolled hypertension, supporting earlier intervention and better cardiovascular disease prevention. Of those receiving treatment, 43.4% had uncontrolled hypertension, which is higher than the global rate of 38.3%, [31] although similar to the Australian rate of 45.7% [23] and whilst BP control did not differ significantly by age in our study, these findings maybe constrained by the sample size.

Exploratory analyses of the relationship between oral health and hypertension found no association between markers of poor oral hygiene including tooth brushing and interdental cleaning and hypertension. Previous findings from a meta-analysis reported a higher risk of hypertension in those with a lower frequency of tooth brushing [10]. However, of the eight studies in this meta-analysis, five were not adjusted for confounders, and the majority were cross- sectional studies. Accordingly, further well‑designed studies with larger sample sizes are needed to examine these associations.

Gingival bleeding has been identified as a marker that is consistently associated with hypertension [32]. We did not objectively assess gingival bleeding and although the reduced frequency of tooth brushing may be considered a surrogate marker for gingival bleeding it is important to note that over 90% of our population brushed once a day or more. Therefore, the difference in brushing between the group with a higher compared to a lower frequency of brushing may not have had a clinically significant impact on gingival bleeding. Periodontal treatment needs as measured by PSR, were not associated with hypertension in our study. Previous studies reported an association between periodontitis and hypertension [11]. Although PSR is an effective screening tool for periodontitis, it is not a diagnostic tool and, therefore, may not be an effective marker of hypertension risk. Significant tooth loss was associated with hypertension in the unadjusted model; however, after adjusting for age, sex, T2DM and smoking status no association was evident. This finding is consistent with global data that have shown that although tooth loss is associated with hypertension in younger adults, the association is not evident in adults over the age of 60 years [33].

The key strength of this study is that it is the first to screen an Australian population visiting a public dental clinic for both heart and oral disease risk factors. Previous studies in North America and Europe have supported the use of dental clinics as a suitable setting for screening and referring patients with non-communicable diseases (NCDs) like heart disease and diabetes [34,35]. This study extends this concept to the Australian context and highlights the shared risk factors between oral diseases and NCDs. The study also demonstrates the feasibility of integrating health screenings into the routine workflow of dental clinics without significantly disrupting patient care. This serves as a proof of concept that could be scaled and adapted in other dental settings

A limitation of this study is that it was conducted at a single site with a convenience sample, this limits the generalisability of the findings to the broader population. Additionally, the overall recruitment of 54% may have introduced selection bias which could over- or underestimate the prevalence of hypertension. However, this study does enable an in-depth understanding of the specific population attending public dental clinics in Western Sydney. Furthermore, although the survey was only available in English, the sample reflects the diversity of the population in Western Sydney (>70% speak a language other than English) and provides valuable insights into the health profiles of a population with a high burden of both oral and other chronic diseases, which could be crucial for designing targeted interventions and policies for similar populations in other regions. Use of a self-report survey to collect oral health information introduces the potential for information bias as participants may under- or over-report health information such as tooth brushing and flossing frequency.

Another limitation was that due to time constraints only two BP measurements were collected per participant instead of the recommended three, which may have led to an overestimation of true BP levels. However, the study’s BP assessments were conducted in a real-world dental clinic setting, which, despite this limitation and the potential for white-coat hypertension, reflects the practical challenges and realities of integrating hypertension screening into routine dental care.

## Conclusion

The proportion with hypertension as determined in this study was higher than the national average, with lower levels of awareness and treatment among younger compared to older adults. These findings underscore the need to improve hypertension awareness and management across adult age groups but particularly in younger adults who may have fewer interactions with medical services. Dental settings may provide a potential venue for opportunistic BP measurement in a population with a higher prevalence of hypertension.

## Supporting information

S1 Table Characteristics of study population by smaller age groups. Values are shown as *n (%)* for categorical variables and *mean ± standard deviation* for continuous variables.
(DOCX)

S1 Checklist STROBE check list_ScreenMe. (DOC)
