# Supplementary material for: Screening for hypertension in a public dental clinic: A single-centre cross-sectional study in Australia
Source: PLoS One. 2026 Jun 11;21(6):e0351629. doi: 10.1371/journal.pone.0351629 (PMC13258008; doi:10.1371/journal.pone.0351629)
Supplement: S1 Table — Values are shown as n (%) for categorical variables and mean ± standard deviation for continuous variables. (DOCX) [file pone.0351629.s001.docx]

**Supplementary table 1: Characteristics of study population by smaller age groups**

| **Characteristic** | **18-24**, N = 10*^1^* | **25-34**, N = 24*^1^* | **35-44**, N = 38*^1^* | **45-54**, N = 32*^1^* | **55-64**, N = 42*^1^* | **65-74**, N = 93*^1^* | **75-84**, N = 51*^1^* | **85-91**, N = 11*^1^* |
| --- | --- | --- | --- | --- | --- | --- | --- | --- |
| **Demographic characteristics** | | | | | | | | |
| **Sex** |  | | | | | | | |
| - Male | 1 / 9 (11.1%) | 6 / 24 (25.0%) | 18 / 37 (48.6%) | 8 / 32 (25.0%) | 16 / 42 (38.1%) | 40 / 91 (44.0%) | 20 / 51 (39.2%) | 7 / 11 (63.6%) |
| - Female | 8 / 9 (88.9%) | 18 / 24 (75.0%) | 19 / 37 (51.4%) | 24 / 32 (75.0%) | 26 / 42 (61.9%) | 51 / 91 (56.0%) | 31 / 51 (60.8%) | 4 / 11 (36.4%) |
| **Speak a language other than English** | 6 / 10 (60.0%) | 12 / 24 (50.0%) | 27 / 38 (71.1%) | 21 / 32 (65.6%) | 33 / 42 (78.6%) | 73 / 93 (78.5%) | 38 / 51 (74.5%) | 7 / 11 (63.6%) |
| **Aboriginal or Torres Strait Islander origin** | 1 / 9 (11.1%) | 3 / 23 (13.0%) | 1 / 37 (2.7%) | 1 / 31 (3.2%) | 1 / 42 (2.4%) | 1 / 92 (1.1%) | 0 / 51 (0.0%) | 0 / 11 (0.0%) |
| **Income** |  |  |  |  |  |  |  |  |
| Dont know | 0 / 10 (0.0%) | 0 / 24 (0.0%) | 0 / 38 (0.0%) | 0 / 32 (0.0%) | 0 / 42 (0.0%) | 2 / 92 (2.2%) | 0 / 51 (0.0%) | 1 / 11 (9.1%) |
| - < $300/w ($16,000/y) | 5 / 10 (50.0%) | 6 / 24 (25.0%) | 6 / 38 (15.8%) | 3 / 32 (9.4%) | 6 / 42 (14.3%) | 6 / 92 (6.5%) | 0 / 51 (0.0%) | 0 / 11 (0.0%) |
| - $300-$999/w ($16,000-$51,999/y) | 4 / 10 (40.0%) | 17 / 24 (70.8%) | 29 / 38 (76.3%) | 26 / 32 (81.3%) | 35 / 42 (83.3%) | 82 / 92 (89.1%) | 50 / 51 (98.0%) | 10 / 11 (90.9%) |
| - ≥ $1000 (≥ $52,000/y) | 0 / 10 (0.0%) | 1 / 24 (4.2%) | 2 / 38 (5.3%) | 3 / 32 (9.4%) | 1 / 42 (2.4%) | 1 / 92 (1.1%) | 0 / 51 (0.0%) | 0 / 11 (0.0%) |
| - Prefer not to say | 1 / 10 (10.0%) | 0 / 24 (0.0%) | 1 / 38 (2.6%) | 0 / 32 (0.0%) | 0 / 42 (0.0%) | 1 / 92 (1.1%) | 1 / 51 (2.0%) | 0 / 11 (0.0%) |
| **Highest level of education** |  | | | | | | | |
| - Primary School | 0 / 10 (0.0%) | 1 / 24 (4.2%) | 1 / 38 (2.6%) | 2 / 32 (6.3%) | 4 / 42 (9.5%) | 13 / 93 (14.0%) | 13 / 51 (25.5%) | 1 / 10 (10.0%) |
| - Secondary School | 6 / 10 (60.0%) | 12 / 24 (50.0%) | 19 / 38 (50.0%) | 12 / 32 (37.5%) | 24 / 42 (57.1%) | 47 / 93 (50.5%) | 20 / 51 (39.2%) | 8 / 10 (80.0%) |
| - Certificate/diploma/TAFE | 2 / 10 (20.0%) | 7 / 24 (29.2%) | 10 / 38 (26.3%) | 9 / 32 (28.1%) | 6 / 42 (14.3%) | 11 / 93 (11.8%) | 9 / 51 (17.6%) | 1 / 10 (10.0%) |
| - University | 2 / 10 (20.0%) | 4 / 24 (16.7%) | 8 / 38 (21.1%) | 9 / 32 (28.1%) | 8 / 42 (19.0%) | 22 / 93 (23.7%) | 9 / 51 (17.6%) | 0 / 10 (0.0%) |
| **Health service utilisation** | | | | | | | | |
| **Last medical check up** |  | | | | | | | |
| - ≥ 1 year ago | 5/10  (50.0%) | 5/24  (20.9%) | 10/38  (26.4%) | 5/32  (15.7%) | 9/42  (21.4%) | 6/93  (6.5%) | 4 / 51 (7.8%) | 0 / 11 (0.0%) |
| - Less than 12 months ago | 5 / 10 (50.0%) | 19 / 24 (79.2%) | 28 / 38 (73.7%) | 27 / 32 (84.4%) | 33 / 42 (78.6%) | 87 / 93 (93.5%) | 47 / 51 (92.2%) | 11 / 11 (100.0%) |
| **Last blood pressure check** |  | | | | | | | |
| - ≥ 1 year ago | 7/10  (70.0%) | 8/24  (33.3%) | 10/38  (26.3%) | 4/32  (12.6%) | 5/42  (11.9%) | 6/93  (6.5%) | 2 / 51 (3.9%) | 0 / 11 (0.0%) |
| Less than 12 months ago | 3 / 10 (30.0%) | 16 / 24 (66.7%) | 28 / 38 (73.7%) | 28 / 32 (87.5%) | 37 / 42 (88.1%) | 87 / 93 (93.5%) | 49 / 51 (96.1%) | 11 / 11 (100.0%) |
| **Last cholesterol check** |  | | | | | | | |
| - ≥ 1 year ago | 5/10  (50.0%) | 6/24  (25.0%) | 12/38  (31.6%) | 9/32  (28.2%) | 8/42  (19.0%) | 9/93  (9.7%) | 4 / 51 (7.8%) | 1 / 11 (9.1%) |
| - Less than 12 months ago | 5 / 10 (50.0%) | 18 / 24 (75.0%) | 26 / 38 (68.4%) | 23 / 32 (71.8%) | 34 / 42 (81.0%) | 84 / 93 (90.3%) | 47 / 51 (92.2%) | 10 / 11 (90.9%) |
| **Last blood glucose test** |  | | | | | | | |
| - ≥ 1 year ago | 5/10  (50.0%) | 6/24  (25.0%) | 11/38  (28.9%) | 9/32  (28.2%) | 8/42  (19.0%) | 9/93  (9.7%) | 4 / 51 (7.8%) | 2 / 11 (18.2%) |
| - Less than 12 months ago | 5 / 10 (50.0%) | 18 / 24 (75.0%) | 27 / 38 (71.1%) | 23 / 32 (71.8%) | 34 / 42 (81.0%) | 83 / 92 (90.2%) | 47 / 51 (92.2%) | 9 / 11 (81.8%) |
| **Cardiovascular Health** | | | | | | | | |
| **BMI** | 26.9 (5.9) | 27.7 (7.7) | 28.4 (5.8) | 32.5 (7.6) | 30.3 (6.1) | 29.4 (6.0) | 28.7 (5.1) | 24.6 (4.1) |
| **Systolic blood pressure (mm/hg)** | 106.6 (11.7) | 111.1 (11.9) | 116.9 (14.5) | 124.3 (14.2) | 129.7 (20.6) | 137.3 (19.8) | 130.4 (16.8) | 133.8 (16.4) |
| **Diastolic blood pressure (mm/hg)** | 68.3 (6.7) | 73.5 (8.3) | 76.4 (10.5) | 77.8 (10.5) | 78.7 (11.7) | 75.3 (11.3) | 70.6 (11.1) | 62.5 (13.5) |
| **High cholesterol** | 1 / 10 (10.0%) | 2 / 24 (8.3%) | 6 / 37 (16.2%) | 13 / 32 (40.6%) | 19 / 41 (46.3%) | 58 / 93 (62.4%) | 34 / 50 (68.0%) | 6 / 11 (54.5%) |
| **Medication to lower your cholesterol** | 0 / 1 (0.0%) | 0 / 2 (0.0%) | 3 / 6 (50.0%) | 8 / 13 (61.5%) | 16 / 19 (84.2%) | 51 / 58 (87.9%) | 31 / 33 (93.9%) | 6 / 6 (100.0%) |
| **Stroke or transient ischaemic attack (TIA)** | 0 / 10 (0.0%) | 0 / 24 (0.0%) | 0 / 36 (0.0%) | 1 / 32 (3.1%) | 1 / 40 (2.5%) | 6 / 93 (6.5%) | 2 / 50 (4.0%) | 0 / 11 (0.0%) |
| **Heart attack** | 0 /10  (0%) | 0 /23  (0%) | 0 / 36 (0%) | 0 / 32  (0%) | 1 / 41 (2.4%) | 5/93  (5.4%) | 5/51 (9.8%) | 1/ 11 (9.1%) |
| **Type 2 Diabetes Mellitus** | 0 / 10 (0.0%) | 0 / 23 (0.0%) | 3 / 38 (7.9%) | 5 / 32 (15.6%) | 7 / 37 (18.9%) | 31 / 92 (33.7%) | 14 / 47 (29.8%) | 1 / 9 (11.1%) |
| **Atrial fibrillation** | 0 / 10 (0.0%) | 1 / 24 (4.2%) | 0 / 38 (0.0%) | 3 / 32 (9.4%) | 1 / 42 (2.4%) | 6 / 93 (6.5%) | 3 / 51 (5.9%) | 1 / 11 (9.1%) |
| **Smoking status** |  | | | | | | | |
| - Never smoked | 8 / 10 (80.0%) | 17 / 24 (70.8%) | 15 / 38 (39.5%) | 16 / 32 (50.0%) | 19 / 42 (45.2%) | 55 / 92 (59.8%) | 31 / 51 (60.8%) | 3 / 11 (27.3%) |
| - Previously smoked (stopped more than 1 year ago) | 1 / 10 (10.0%) | 3 / 24 (12.5%) | 3 / 38 (7.9%) | 8 / 32 (25.0%) | 9 / 42 (21.4%) | 25 / 92 (27.2%) | 19 / 51 (37.3%) | 8 / 11 (72.7%) |
| - Currently smoke (or stopped less than or equal to 1 year ago) | 1 / 10 (10.0%) | 4 / 24 (16.7%) | 20 / 38 (52.6%) | 8 / 32 (25.0%) | 14 / 42 (33.3%) | 12 / 92 (13.0%) | 1 / 51 (2.0%) | 0 / 11 (0.0%) |
| **Oral Health** | | | | | | | | |
| **Tooth brushing frequency** |  | | | | | | | |
| - Less than once a day | 0 / 10 (0.0%) | 1 / 24 (4.2%) | 8 / 37 (21.6%) | 3 / 32 (9.4%) | 4 / 42 (9.5%) | 2 / 93 (2.2%) | 2 / 51 (3.9%) | 2 / 11 (18.2%) |
| - Once a day | 5 / 10 (50.0%) | 10 / 24 (41.7%) | 17 / 37 (45.9%) | 18 / 32 (56.3%) | 17 / 42 (40.5%) | 44 / 93 (47.3%) | 22 / 51 (43.1%) | 3 / 11 (27.3%) |
| - Twice or more times per day | 5 / 10 (50.0%) | 13 / 24 (54.2%) | 12 / 37 (32.4%) | 11 / 32 (34.4%) | 21 / 42 (50.0%) | 47 / 93 (50.5%) | 27 / 51 (52.9%) | 6 / 11 (54.5%) |
| **Use floss or interdental brushes** | 6 / 10 (60.0%) | 12 / 24 (50.0%) | 21 / 37 (56.8%) | 18 / 32 (56.3%) | 25 / 41 (61.0%) | 54 / 92 (58.7%) | 28 / 49 (57.1%) | 2 / 10 (20.0%) |
| **Drink fluoridated tap water** | 8 / 10 (80.0%) | 15 / 23 (65.2%) | 35 / 38 (92.1%) | 21 / 32 (65.6%) | 35 / 42 (83.3%) | 80 / 93 (86.0%) | 43 / 51 (84.3%) | 10 / 11 (90.9%) |
| **Use a standard fluoride containing tooth paste at least once a day** | 10 / 10 (100.0%) | 22 / 24 (91.7%) | 35 / 37 (94.6%) | 30 / 32 (93.8%) | 39 / 42 (92.9%) | 87 / 89 (97.8%) | 48 / 50 (96.0%) | 9 / 11 (81.8%) |
| **Drink at least 2L of water every day** | 7 / 10 (70.0%) | 11 / 24 (45.8%) | 16 / 38 (42.1%) | 11 / 32 (34.4%) | 22 / 42 (52.4%) | 39 / 93 (41.9%) | 19 / 51 (37.3%) | 5 / 11 (45.5%) |
| **Add sugar to tea/coffee** | 1 / 10 (10.0%) | 12 / 24 (50.0%) | 24 / 38 (63.2%) | 16 / 32 (50.0%) | 24 / 41 (58.5%) | 45 / 91 (49.5%) | 26 / 51 (51.0%) | 5 / 11 (45.5%) |
| **Sugar sweetened beverages** |  | | | | | | | |
| - Twice or more times per day, | 0 / 10 (0.0%) | 5 / 24 (20.8%) | 9 / 38 (23.7%) | 5 / 32 (15.6%) | 7 / 42 (16.7%) | 7 / 92 (7.6%) | 4 / 51 (7.8%) | 2 / 11 (18.2%) |
| - Once a day | 0 / 10 (0.0%) | 6 / 24 (25.0%) | 6 / 38 (15.8%) | 5 / 32 (15.6%) | 2 / 42 (4.8%) | 8 / 92 (8.7%) | 6 / 51 (11.8%) | 0 / 11 (0.0%) |
| - I dont drink sugar sweetened beverages | 1 / 10 (10.0%) | 6 / 24 (25.0%) | 7 / 38 (18.4%) | 7 / 32 (21.9%) | 14 / 42 (33.3%) | 36 / 92 (39.1%) | 21 / 51 (41.2%) | 3 / 11 (27.3%) |
| - Less than once a day | 9 / 10 (90.0%) | 7 / 24 (29.2%) | 16 / 38 (42.1%) | 15 / 32 (46.9%) | 19 / 42 (45.2%) | 41 / 92 (44.6%) | 20 / 51 (39.2%) | 6 / 11 (54.5%) |
| **Snack frequently (more than 3 times a day)** | 7 / 10 (70.0%) | 8 / 24 (33.3%) | 15 / 38 (39.5%) | 11 / 32 (34.4%) | 11 / 42 (26.2%) | 22 / 91 (24.2%) | 8 / 51 (15.7%) | 1 / 11 (9.1%) |
| **Medications that make your mouth dry** | 2 / 10 (20.0%) | 10 / 23 (43.5%) | 9 / 37 (24.3%) | 12 / 32 (37.5%) | 22 / 40 (55.0%) | 41 / 92 (44.6%) | 19 / 51 (37.3%) | 8 / 11 (72.7%) |
| **Number of teeth present (assessment form)** |  | | | | | | | |
| - Number of teeth (mean) | 29.7 (2.9) | 27.1 (6.0) | 25.9 (6.5) | 24.8 (4.8) | 21.5 (8.7) | 19.0 (8.4) | 17.4 (9.2) | 7.9  (8.3) |
| - No teeth | 0 / 10 (0.0%) | 0 / 24 (0.0%) | 1 / 38 (2.6%) | 0 / 32 (0.0%) | 3 / 42 (7.1%) | 6 / 93 (6.5%) | 1 / 51 (2.0%) | 2 / 11 (18.2%) |
| - 1-9 teeth | 0 / 10 (0.0%) | 1 / 24 (4.2%) | 1 / 38 (2.6%) | 0 / 32 (0.0%) | 2 / 42 (4.8%) | 9 / 93 (9.7%) | 14 / 51 (27.5%) | 5 / 11 (45.5%) |
| - 10-19 teeth | 0 / 10 (0.0%) | 0 / 24 (0.0%) | 1 / 38 (2.6%) | 5 / 32 (15.6%) | 10 / 42 (23.8%) | 27 / 93 (29.0%) | 10 / 51 (19.6%) | 2 / 11 (18.2%) |
| - >20 teeth | 10 / 10 (100.0%) | 23 / 24 (95.8%) | 35 / 38 (92.1%) | 27 / 32 (84.4%) | 27 / 42 (64.3%) | 51 / 93 (54.8%) | 26 / 51 (51.0%) | 2 / 11 (18.2%) |
| **Number of teeth (self reported)** | 31.3 (1.6) | 28.4 (6.1) | 26.6 (6.6) | 25.0 (6.0) | 21.6 (9.4) | 19.6 (9.4) | 17.5 (10.1) | 9.9 (11.0) |
| **Number of fillings needed** | 0.9 (1.3) | 2.5 (2.6) | 2.8 (3.0) | 1.9 (1.9) | 1.4 (2.5) | 1.8 (2.8) | 1.3 (1.9) | 1.3 (1.3) |
| **PSR code** |  | | | | | | | |
| - 0-2 | 5 / 10 (50.0%) | 13 / 24 (54.2%) | 19 / 37 (51.4%) | 13 / 30 (43.3%) | 23 / 40 (57.5%) | 42 / 86 (48.8%) | 25 / 50 (50.0%) | 4 / 9 (44.4%) |
| - 3-4 | 5 / 10 (50.0%) | 11 / 24 (45.8%) | 18 / 37 (48.6%) | 17 / 30 (56.7%) | 17 / 40 (42.5%) | 44 / 86 (51.2%) | 25 / 50 (50.0%) | 5 / 9 (55.6%) |
| **Lifestyle Factors** | | | | | | | | |
| **Alcohol** |  |  |  |  |  |  |  |  |
| - Never | 6 / 10 (60.0%) | 13 / 24 (54.2%) | 23 / 38 (60.5%) | 18 / 31 (58.1%) | 27 / 42 (64.3%) | 57 / 92 (62.0%) | 23 / 50 (46.0%) | 5 / 11 (45.5%) |
| - Less than or equal to 2 standard drinks a day | 4 / 10 (40.0%) | 9 / 24 (37.5%) | 13 / 38 (34.2%) | 11 / 31 (35.5%) | 13 / 42 (31.0%) | 33 / 92 (35.9%) | 27 / 50 (54.0%) | 6 / 11 (54.5%) |
| - More than 2 standard drinks a day | 0 / 10 (0.0%) | 2 / 24 (8.3%) | 2 / 38 (5.3%) | 2 / 31 (6.5%) | 2 / 42 (4.8%) | 2 / 92 (2.2%) | 0 / 50 (0.0%) | 0 / 11 (0.0%) |
| **Servings of vegetables** |  |  |  |  |  |  |  |  |
| - None | 0 / 10 (0.0%) | 3 / 24 (12.5%) | 0 / 38 (0.0%) | 2 / 32 (6.3%) | 1 / 42 (2.4%) | 7 / 93 (7.5%) | 0 / 51 (0.0%) | 0 / 11 (0.0%) |
| - 1-4 serves daily | 8 / 10 (80.0%) | 13 / 24 (54.2%) | 21 / 38 (55.3%) | 20 / 32 (62.5%) | 25 / 42 (59.5%) | 68 / 93 (73.1%) | 44 / 51 (86.3%) | 6 / 11 (54.5%) |
| - 5 or more serves of vegetables per day | 0 / 10 (0.0%) | 1 / 24 (4.2%) | 4 / 38 (10.5%) | 2 / 32 (6.3%) | 9 / 42 (21.4%) | 6 / 93 (6.5%) | 4 / 51 (7.8%) | 3 / 11 (27.3%) |
| - I dont eat vegetables everyday | 2 / 10 (20.0%) | 7 / 24 (29.2%) | 13 / 38 (34.2%) | 8 / 32 (25.0%) | 7 / 42 (16.7%) | 12 / 93 (12.9%) | 3 / 51 (5.9%) | 2 / 11 (18.2%) |
| **Servings of fruit** |  |  |  |  |  |  |  |  |
| - None | 4 / 10 (40.0%) | 13 / 24 (54.2%) | 19 / 38 (50.0%) | 15 / 32 (46.9%) | 7 / 42 (16.7%) | 20 / 93 (21.5%) | 9 / 51 (17.6%) | 4 / 11 (36.4%) |
| - 1 serve a day | 4 / 10 (40.0%) | 6 / 24 (25.0%) | 10 / 38 (26.3%) | 9 / 32 (28.1%) | 15 / 42 (35.7%) | 25 / 93 (26.9%) | 13 / 51 (25.5%) | 4 / 11 (36.4%) |
| - 2 or more serves a day | 2 / 10 (20.0%) | 5 / 24 (20.8%) | 9 / 38 (23.7%) | 8 / 32 (25.0%) | 20 / 42 (47.6%) | 48 / 93 (51.6%) | 29 / 51 (56.9%) | 3 / 11 (27.3%) |
| **Exercise** |  |  |  |  |  |  |  |  |
| - Not at all | 5 / 10 (50.0%) | 11 / 23 (47.8%) | 22 / 36 (61.1%) | 16 / 31 (51.6%) | 16 / 41 (39.0%) | 48 / 91 (52.7%) | 25 / 50 (50.0%) | 5 / 11 (45.5%) |
| - Less than 30 minutes 5 days a week | 1 / 10 (10.0%) | 2 / 23 (8.7%) | 2 / 36 (5.6%) | 6 / 31 (19.4%) | 5 / 41 (12.2%) | 11 / 91 (12.1%) | 6 / 50 (12.0%) | 3 / 11 (27.3%) |
| - 30 minutes or more 5 days a week | 4 / 10 (40.0%) | 10 / 23 (43.5%) | 12 / 36 (33.3%) | 9 / 31 (29.0%) | 20 / 41 (48.8%) | 32 / 91 (35.2%) | 19 / 50 (38.0%) | 3 / 11 (27.3%) |
| *^1^* n / N (%); Mean (SD) | | | | | | | | |
