# Supplementary material for: Screening for hypertension in a public dental clinic: A single-centre cross-sectional study in Australia
Source: PLoS One. 2026 Jun 11;21(6):e0351629. doi: 10.1371/journal.pone.0351629 (PMC13258008; doi:10.1371/journal.pone.0351629)
Supplement: Attachment — Submitted filename: PLOS10825.docx [file pone.0351629.s003.docx]

1. The article is very well written but needs a stronger statistical corroboration.
2. Methodology of BP recording: Reason for two rather than the recommended three office BP measurements. This is a confounder.
3. The sample size was meant to detect hypertension. There was no provision for analysis of the correlation between dental problems and HTN and the relevance of the same. The sample size was for a single cohort. But the analysis was for two cohorts.
4. Overall recruitment rate of 54% will have a selection bias – this may not be a truly representative population. May inflate or deflate the prevalence.
5. When two groups are mentioned in Table 1 – the p value for the differences are not mentioned. This is important to know the baseline difference between the groups. Table 2 also needs similar p values. The study was not powered for these analyses, but these are done.
6. Hypertension prevalence increases with age, and this is quite striking. Needs mention
7. Para 1 of discussion – two inferences – the overall treatment and awareness percentages? without difference in age and the poor treatment and control among younger individuals needs statistical correlation.
8. The dental correlation is underpowered.
9. Limitation – underpowered to correlate the oral pathology with hypertension. 54% recruitment has to be justified. Confounders need clear mention - the role of pain and its impact on the control, two measurements rather than three that can falsely elevate BP.
10. The statement that screening among younger is more beneficial needs stress

Overall needs stronger statistical backup. The numbers are not sufficient when two groups are analyzed and may need additional meetings with the statistician to make the paper stronger.
