# Supplementary material for: Screening for hypertension in a public dental clinic: A single-centre cross-sectional study in Australia
Source: PLoS One. 2026 Jun 11;21(6):e0351629. doi: 10.1371/journal.pone.0351629 (PMC13258008; doi:10.1371/journal.pone.0351629)
Supplement: Attachment — Submitted filename: Response to reviewers.docx [file pone.0351629.s005.docx]

A/Prof Shalinie King

Sydney Dental School

The University of Sydney

Editor Plos One

Date 1^st^ May 2026

Dear Editor in Chief,

Thank you for taking the time to review this manuscript. Please see responses to journal requirements below:

**Journal Requirements**:

When submitting your revision, we need you to address these additional requirements.

1.Please ensure that your manuscript meets PLOS ONE's style requirements, including those for file naming. The PLOS ONE style templates can be found at

[https://journals.plos.org/plosone/s/file?id=wjVg/PLOSOne_formatting_sample_main_body.pdf](https://url.au.m.mimecastprotect.com/s/y851C71R2NTQyMLQ9iNs1To4xJg?domain=journals.plos.org) and

[https://journals.plos.org/plosone/s/file?id=ba62/PLOSOne_formatting_sample_title_authors_affiliations.pdf](https://url.au.m.mimecastprotect.com/s/-nxaC81V0PTzQ39zxhotQTyc6hZ?domain=track.editorialmanager.com)

**Response**: The manuscript has been formatted according to the specified requirements.

2. Thank you for stating the following financial disclosure:

“The project was funded by a research grant from the Charles Perkins Centre, The University of Sydney”

Please state what role the funders took in the study.  If the funders had no role, please state: "The funders had no role in study design, data collection and analysis, decision to publish, or preparation of the manuscript."

If this statement is not correct you must amend it as needed.

Please include this amended Role of Funder statement in your cover letter; we will change the online submission form on your behalf.

**Response**: We can confirm that “The funders had no role in study design, data collection and analysis, decision to publish, or preparation of the manuscript."

3. If the reviewer comments include a recommendation to cite specific previously published works, please review and evaluate these publications to determine whether they are relevant and should be cited. There is no requirement to cite these works unless the editor has indicated otherwise.

We have addressed each reviewer comment below:

**Reviewer #1**

This cross-sectional study by King et al. investigates the relationship between blood pressure and markers of poor oral health among adults aged ≥18 years attending dental clinics in Australia. Although the authors state that this is the first report using Australian data, several concerns should be addressed.

**1.** In terms of study population and generalizability, the most significant concern is that this study was conducted in a single center and included individuals attending facilities that primarily provide care for low-income populations. While the authors emphasize that this is the first study of its kind in Australia, inclusion of patients from general dental clinics would likely provide more robust and generalizable results.

**Response**: We agree and this is a limitation of the study. To make this more explicit we have revised the limitations section and included the following wording in the Discussion:

*A limitation of this study is that it was conducted at a single site with a convenience sample,* ***this limits the generalisability of the findings to the broader population.***

We do already acknowledge that this is an at-risk population and have noted in the Discussion that this study:

*provides valuable insights into the health profiles of a population with a high burden of both oral and other chronic diseases, which could be crucial for designing targeted interventions and policies for similar populations in other regions.*

**2.** As noted in both the Methods and Discussion sections, there are concerns regarding the method of blood pressure measurement. However, considering the authors’ point that these measurements reflect real-world practice in dental clinics, it may be more appropriate to revise the definition of “Hypertension” in Table 2 to “Determined hypertension (diagnosed and/or treated) and high measured blood pressure.”
This is because even if the measurement method does not strictly follow guideline-recommended procedures, repeatedly elevated blood pressure readings obtained incidentally may indicate either true hypertension or elevated blood pressure, representing a population at increased risk for future hypertension. (Curr Hypertens Rep. 2012:14:619-25. doi:10.1007/s11906-012-0299-y, BMC Cardiovasc Disord. 2021: 21:523. doi: 10.1186/s12872-021-02334-6.).

**Response**: Thank you for highlighting this. We have revised the definition of Hypertension in table 2, where we have included a summary of the definition within the table. We have also clarified this in the conclusion.

Discussion: *Hypertension as determined in this study was prevalent in this cohort, with lower levels of awareness and treatment among younger compared to older adults.*

**3.** On page 18, regarding hypertension awareness, it would be informative to present the proportion of individuals who are aware of having hypertension or elevated blood pressure and are receiving treatment (or, alternatively, not receiving treatment).

**Response**: All the people who were treated with anti-hypertensives were aware of their diagnosis. Therefore, the people who are presented in table 2 as being treated are also aware. We have reworded the table to make that clear.

**4.** In terms of interpretation of the conclusion, the conclusion that “Oral health practitioners could play an important role in primary care by screening younger adults with significant oral disease for undiagnosed or uncontrolled hypertension” is not entirely convincing based on the current data. Further clarification is needed to justify this statement.

**Response**: we have reworded the **abstract conclusion** and **manuscript conclusion** to better reflect the data –

***Abstract****: In this population with a high burden of oral disease, hypertension was common highlighting the importance of opportunistic blood pressure assessment. Dental settings may offer an additional point of contact for identifying individuals with undiagnosed or uncontrolled hypertension, particularly among younger adults with lower awareness.*

***Manuscript****: Hypertension as determined in this study was prevalent in this cohort, with lower levels of awareness and treatment among younger compared to older adults. These findings underscore the need to improve hypertension awareness and management across adult age groups but particularly in younger adults who may have fewer interactions with medical services. Dental settings may provide a potential venue for opportunistic BP measurement.*

**Reviewer #2**

The article is very well written but needs a stronger statistical corroboration.
**1.** Methodology of BP recording: Reason for two rather than the recommended three office BP measurements. This is a confounder.

**Response**: We agree that it would have been more appropriate to record three blood pressure recordings, however due to limitations in time in a real world clinical setting it was only feasible to record two readings. We have acknowledged this as a limitation and added to the discussion –

Discussion:

*Another limitation was that due to time constraints only two BP measurements were collected per participant instead of the recommended three, which may have led to an overestimation of true BP levels. However, the study’s BP assessments were conducted in a real-world dental clinic setting …*

**2.** The sample size was meant to detect hypertension. There was no provision for analysis of the correlation between dental problems and HTN and the relevance of the same. The sample size was for a single cohort. But the analysis was for two cohorts.

**Response**: Thank you, we acknowledge that this study was not powered to detect differences between age cohorts and to identify associations between markers of oral health and hypertension. However, the exploratory analysis did detect significant differences in hypertension prevalence, awareness and treatment between the two age cohorts. We therefore felt it was important to present this. We have now included p-values to support these findings. We have also revised the **discussion**.

Discussion*: Similarly, despite the small sample size, we found that younger individuals were more likely than older individuals to be unaware of their condition, and untreated (29.3% vs 13.2%, 43.9% vs 15.8%, respectively). Hence, screening patients in Australian public dental clinics, especially younger adults is an opportunity to identify and refer those with undiagnosed or uncontrolled hypertension, supporting earlier intervention and better cardiovascular disease prevention. Of those receiving treatment, 43.4% had uncontrolled hypertension, which is higher than the global rate of 38.3%,[31] although similar to the Australian rate of 45.7% [23] and whilst BP control did not differ significantly by age in our study, these findings maybe constrained by the sample size.*

We also acknowledge that the finding that there was no association between markers of oral health and HTN may relate to the fact that the study was underpowered and have reworded sections of the **discussion** as outlined below:

Discussion [First paragraph]: *The exploratory analysis did not identify a significant association between markers of poor oral hygiene, significant tooth loss or periodontal treatment needs and hypertension.*

Discussion: *Exploratory analyses of the relationship between oral health and hypertension found no the association between markers of poor oral hygiene including tooth brushing and interdental cleaning and hypertension. Previous findings from of a meta-analysis that reported a higher risk of hypertension in those with a lower frequency of tooth brushing [10]. However, of the eight studies in this meta-analysis, five were not adjusted for confounders, and the majority were cross- sectional studies. Accordingly, further well designed studies with larger sample sizes are needed to examine these associations.*

We have also updated the **objective in the abstract** and the **aims as stated in the introduction** to clarify this:

*Objective: This study aimed to determine hypertension awareness, treatment and control, among adults attending a public dental clinic, and explore age-related differences between younger (< 65 years) and older adults (≥ 65 years) including associations between markers of oral health and hypertension.*

*Introduction (Aims): The aim of this study was to determine hypertension awareness, treatment and control, among adults attending a public dental service, and to explore age‑specific patterns and associations with oral health.*

**3.** Overall recruitment rate of 54% will have a selection bias – this may not be a truly representative population. May inflate or deflate the prevalence.

**Response**: We agree and have included this as a limitation

*Additionally, the overall recruitment of 54% may have introduced selection bias which could over- or underestimate the prevalence of hypertension.*

We have also updated the **results** section to clarify that individuals in pain were excluded (this is already noted in figure 1 but we had omitted this from the text in the results):

*One of the main reasons for exclusion was the need for an interpreter, other reasons included lack of participant time or interest in participation* ***or if the individual was in pain*** *(Figure 1).*

**4.** When two groups are mentioned in Table 1 – the p value for the differences are not mentioned. This is important to know the baseline difference between the groups. Table 2 also needs similar p values. The study was not powered for these analyses, but these are done

**Response**: The p-values have been added to table 1 and table 2 and additional text has been included in the statistical analysis section:

*Differences in proportions between groups were calculated using a chi-square test.*

**5.** Hypertension prevalence increases with age, and this is quite striking. Needs mention

**Response**: Thank you for drawing our attention to this, we have already mentioned this finding in the results and have revised the first paragraph of the discussion to highlight the key findings:

Discussion: *A higher proportion of older (74.1%) compared to younger adults (28.1%) in this population had hypertension and while overall rates of awareness and treatment were high, younger adults were less likely than older adults to be aware and treated for hypertension (56.1% vs 84.2%, 30.0% vs 47.4%, respectively). These findings suggest that within public dental services there are opportunities to address broader cardiovascular disease prevention goals, especially among younger adults.*

**6.** Para 1 of discussion – two inferences – the overall treatment and awareness percentages? without difference in age and the poor treatment and control among younger individuals needs statistical correlation

**Response**: We have included the p-values in Table 2 which demonstrate that the proportion of adults with hypertension awareness and treatment is significantly lower in younger compared to older adults.

We analysed the untreated numbers as well (see below), however did not include this data in the table as we did not feel they contributed any further information. The presentation of data in Table 2 is aligned with data presentation in the May Measurement Month campaign ref: Carnagarin et al 2021 ( <https://doi.org/10.1093/eurheartj/suab016>).

Additionally, we have reworded the first paragraph of the discussion in response to comment #5 and feel that this has now been addressed.

| **Hypertension (Untreated)**  [Number (%) of patients aware and untreated among those with hypertension, missing=1] | 9/156  (5.8%)  (CI: 3.1%, 10.6%) | 6/23  (26.1%)  (CI: 112.5%, 46.5%) | 3/96  (3.1%)  (CI: 1.1%, 8.8%) | **0.002** |
| --- | --- | --- | --- | --- |

**7.** The dental correlation is underpowered.

**Response**: We have acknowledged this and revised several sections of the manuscript including the Abstract, Introduction and Discussion – please see response to comment #2 for details.

**8.** Limitation – underpowered to correlate the oral pathology with hypertension. 54% recruitment must be justified. Confounders need clear mention - the role of pain and its impact on the control, two measurements rather than three that can falsely elevate BP.

**Response**:

- We have addressed the issue of the study being underpowered in our response to comment #2 .
- We have addressed the 54% response rate (see response to comment #3)
- We have clarified that individuals in pain were excluded (figure 1 and response to comment #3).
- We have addressed the two blood pressure measurements and acknowledged this as a limitation (response to comment # 1)

**10.** The statement that screening among younger is more beneficial needs stress

**Response**: Thank you for this comment, we have revised the first paragraph of the discussion to highlight this (see response to comment #5), we have also revised the conclusion to highlight this point:

*Hypertension as determined in this study was prevalent in this cohort, with lower levels of awareness and treatment among younger compared to older adults. These findings underscore the need to improve hypertension awareness and management across adult age groups but particularly in younger adults who may have fewer interactions with medical services. Dental settings may be a potential venue for opportunistic BP measurement.*

Yours Sincerely

Shalinie King
