# Supplementary material for: Screening for hypertension in a public dental clinic: A single-centre cross-sectional study in Australia
Source: PLoS One. 2026 Jun 11;21(6):e0351629. doi: 10.1371/journal.pone.0351629 (PMC13258008; doi:10.1371/journal.pone.0351629)
Supplement: Attachment — Submitted filename: Response to reviewers 2.docx [file pone.0351629.s006.docx]

A/Prof Shalinie King

Sydney Dental School

The University of Sydney

Editor Plos One

Date 26^st^ May 2026

Dear Editor in Chief,

Thank you for taking the time to review this manuscript. Please see responses to reviewer comments below:

Review Comments to the Author

**Reviewer #1**: Although there are several limitations, the authors have responded appropriately to the reviewers’ questions.

**Reviewer #2**: Overall revised version is done well.

Minor corections -
1. the prevalence of HTN in Australia - 22 to 34% in different portions - the statistical and introduction. may be better to give a range - which is quite acceptable

**Response**: Thank you we have provided a range of 22-34% in the introduction.

2. the total number is 302 but the < 65 and > 65 years category adds upto 301 - if this is corrected then most of the numbers have to be carefully reworked. my not change the conclusion.

**Response**: The number of participants in the age categories does not add up to 302 because one participant did not provide their age, we noted missingness in Table 2 but did not note missingness in Table 1. We have now included missingness for all variables in Table 1 to ensure greater clarity.

3. In the conclusion, it might be good to add that the prevalence of HTN in patients attending the dental clinic is higher and provides a very good opportunity to scren a population with a higher prevalence than the population. This will emphasize the importance of the study.

**Response**: We have revised the conclusion to make this point more clearly –

***The proportion with*** *hypertension as determined in this study* ***was higher than the national average****, with lower levels of awareness and treatment among younger compared to older adults. These findings underscore the need to improve hypertension awareness and management across adult age groups but particularly in younger adults who may have fewer interactions with medical services. Dental settings may provide a potential venue for opportunistic BP measurement* ***in a population with a higher prevalence of hypertension.***

Yours Sincerely

Shalinie King
