# Supplementary material for: Screening for hypertension in a public dental clinic: A single-centre cross-sectional study in Australia
Source: PLoS One. 2026 Jun 11;21(6):e0351629. doi: 10.1371/journal.pone.0351629 (PMC13258008; doi:10.1371/journal.pone.0351629)
Supplement: Peer review history

# Peer review: Decision Letter 0

**DOI:** 10.1371/journal.pone.0351629.r001

**Authors:** Masaki Mogi

14 Apr 2026

PONE-D-26-10825

Screening for hypertension in a public dental clinic: A single-centre cross-sectional study in Australia

PLOS One

Dear Dr.  King,

Thank you for submitting your manuscript to PLOS ONE. After careful consideration, we feel that it has merit but does not fully meet PLOS ONE’s publication criteria as it currently stands. Therefore, we invite you to submit a revised version of the manuscript that addresses the points raised during the review process.

Please submit your revised manuscript by May 29 2026 11:59PM. If you will need more time than this to complete your revisions, please reply to this message or contact the journal office at plosone@plos.org. When you're ready to submit your revision, log on to https://www.editorialmanager.com/pone/ and select the 'Submissions Needing Revision' folder to locate your manuscript file.

Please include the following items when submitting your revised manuscript:

- A letter that responds to each point raised by the academic editor and reviewer(s). You should upload this letter as a separate file labeled 'Response to Reviewers'.
- A marked-up copy of your manuscript that highlights changes made to the original version. You should upload this as a separate file labeled 'Revised Manuscript with Track Changes'.
- An unmarked version of your revised paper without tracked changes. You should upload this as a separate file labeled 'Manuscript'.

If you would like to make changes to your financial disclosure, please include your updated statement in your cover letter. Guidelines for resubmitting your figure files are available below the reviewer comments at the end of this letter.

If applicable, we recommend that you deposit your laboratory protocols in protocols.io to enhance the reproducibility of your results. Protocols.io assigns your protocol its own identifier (DOI) so that it can be cited independently in the future. For instructions see: https://journals.plos.org/plosone/s/submission-guidelines#loc-laboratory-protocols. Additionally, PLOS ONE offers an option for publishing peer-reviewed Lab Protocol articles, which describe protocols hosted on protocols.io. Read more information on sharing protocols at https://plos.org/protocols?utm_medium=editorial-email&utm_source=authorletters&utm_campaign=protocols.

As the corresponding author, your ORCID iD is verified in the submission system and will appear in the published article. PLOS supports the use of ORCID, and we encourage all coauthors to register for an ORCID iD and use it as well. Please encourage your coauthors to verify their ORCID iD within the submission system before final acceptance, as unverified ORCID iDs will not appear in the published article. *Only*   the individual author can complete the verification step; PLOS staff *cannot*   verify ORCID iDs on behalf of authors.

We look forward to receiving your revised manuscript.

Kind regards,

Masaki Mogi

Academic Editor

PLOS One

Journal Requirements:

When submitting your revision, we need you to address these additional requirements.

1.Please ensure that your manuscript meets PLOS ONE's style requirements, including those for file naming. The PLOS ONE style templates can be found at

https://journals.plos.org/plosone/s/file?id=wjVg/PLOSOne_formatting_sample_main_body.pdf and

https://journals.plos.org/plosone/s/file?id=ba62/PLOSOne_formatting_sample_title_authors_affiliations.pdf

2. Thank you for stating the following financial disclosure:

“The project was funded by a research grant from the Charles Perkins Centre, The University of Sydney”

Please state what role the funders took in the study.  If the funders had no role, please state: "The funders had no role in study design, data collection and analysis, decision to publish, or preparation of the manuscript."

If this statement is not correct you must amend it as needed.

Please include this amended Role of Funder statement in your cover letter; we will change the online submission form on your behalf.

3. If the reviewer comments include a recommendation to cite specific previously published works, please review and evaluate these publications to determine whether they are relevant and should be cited. There is no requirement to cite these works unless the editor has indicated otherwise.

Additional Editor Comments:

This paper was peer-reviewed by two expert reviewers, who found the content to be interesting but noted that significant revisions are required in many areas, including methodology and the interpretation of results. However, these revisions are expected to clarify the paper’s main points and enhance its reliability. Please revise the paper based on the reviewers’ constructive comments.

[Note: HTML markup is below. Please do not edit.]

Reviewer's Responses to Questions

**Comments to the Author**

1. Is the manuscript technically sound, and do the data support the conclusions?

The manuscript must describe a technically sound piece of scientific research with data that supports the conclusions. Experiments must have been conducted rigorously, with appropriate controls, replication, and sample sizes. The conclusions must be drawn appropriately based on the data presented.

Reviewer #1: Partly

Reviewer #2: Partly

**********

2. Has the statistical analysis been performed appropriately and rigorously?

Reviewer #1: Yes

Reviewer #2: No

**********

3. Have the authors made all data underlying the findings in their manuscript fully available?

The PLOS Data policy requires authors to make all data underlying the findings described in their manuscript fully available without restriction, with rare exception (please refer to the Data Availability Statement in the manuscript PDF file). The data should be provided as part of the manuscript or its supporting information, or deposited to a public repository. For example, in addition to summary statistics, the data points behind means, medians and variance measures should be available. If there are restrictions on publicly sharing data—e.g. participant privacy or use of data from a third party—those must be specified.

Reviewer #1: Yes

Reviewer #2: Yes

**********

4. Is the manuscript presented in an intelligible fashion and written in standard English?

PLOS ONE does not copyedit accepted manuscripts, so the language in submitted articles must be clear, correct, and unambiguous. Any typographical or grammatical errors should be corrected at revision, so please note any specific errors here.

Reviewer #1: Yes

Reviewer #2: Yes

**********

5. Review Comments to the Author

Please use the space provided to explain your answers to the questions above. You may also include additional comments for the author, including concerns about dual publication, research ethics, or publication ethics. (Please upload your review as an attachment if it exceeds 20,000 characters)

Reviewer #1: This cross-sectional study by King et al. investigates the relationship between blood pressure and markers of poor oral health among adults aged ≥18 years attending dental clinics in Australia. Although the authors state that this is the first report using Australian data, several concerns should be addressed.

1. In terms of study population and generalizability, the most significant concern is that this study was conducted in a single center and included individuals attending facilities that primarily provide care for low-income populations. While the authors emphasize that this is the first study of its kind in Australia, inclusion of patients from general dental clinics would likely provide more robust and generalizable results.

2. As noted in both the Methods and Discussion sections, there are concerns regarding the method of blood pressure measurement. However, considering the authors’ point that these measurements reflect real-world practice in dental clinics, it may be more appropriate to revise the definition of “Hypertension” in Table 2 to “Determined hypertension (diagnosed and/or treated) and high measured blood pressure.”

This is because even if the measurement method does not strictly follow guideline-recommended procedures, repeatedly elevated blood pressure readings obtained incidentally may indicate either true hypertension or elevated blood pressure, representing a population at increased risk for future hypertension. (Curr Hypertens Rep. 2012:14:619-25. doi:10.1007/s11906-012-0299-y, BMC Cardiovasc Disord. 2021: 21:523. doi: 10.1186/s12872-021-02334-6.).

3. On page 18, regarding hypertension awareness, it would be informative to present the proportion of individuals who are aware of having hypertension or elevated blood pressure and are receiving treatment (or, alternatively, not receiving treatment).

4. In terms of interpretation of the conclusion, the conclusion that “Oral health practitioners could play an important role in primary care by screening younger adults with significant oral disease for undiagnosed or uncontrolled hypertension” is not entirely convincing based on the current data. Further clarification is needed to justify this statement.

Reviewer #2: 1. The article is very well written but needs a stronger statistical corroboration.

2. Methodology of BP recording: Reason for two rather than the recommended three office BP measurements. This is a confounder.

3. The sample size was meant to detect hypertension. There was no provision for analysis of the correlation between dental problems and HTN and the relevance of the same. The sample size was for a single cohort. But the analysis was for two cohorts.

4. Overall recruitment rate of 54% will have a selection bias – this may not be a truly representative population. May inflate or deflate the prevalence.

5. When two groups are mentioned in Table 1 – the p value for the differences are not mentioned. This is important to know the baseline difference between the groups. Table 2 also needs similar p values. The study was not powered for these analyses, but these are done.

6. Hypertension prevalence increases with age, and this is quite striking. Needs mention

7. Para 1 of discussion – two inferences – the overall treatment and awareness percentages? without difference in age and the poor treatment and control among younger individuals needs statistical correlation.

8. The dental correlation is underpowered.

9. Limitation – underpowered to correlate the oral pathology with hypertension. 54% recruitment must be justified. Confounders need clear mention - the role of pain and its impact on the control, two measurements rather than three that can falsely elevate BP.

10. The statement that screening among younger is more beneficial needs stress

**********

6. PLOS authors have the option to publish the peer review history of their article (what does this mean?). If published, this will include your full peer review and any attached files.

If you choose “no”, your identity will remain anonymous but your review may still be made public.

**Do you want your identity to be public for this peer review?**   For information about this choice, including consent withdrawal, please see our Privacy Policy.

Reviewer #1: No

Reviewer #2: No

**********

[NOTE: If reviewer comments were submitted as an attachment file, they will be attached to this email and accessible via the submission site. Please log into your account, locate the manuscript record, and check for the action link "View Attachments". If this link does not appear, there are no attachment files.]

To ensure your figures meet our technical requirements, please review our figure guidelines: https://journals.plos.org/plosone/s/figures

You may also use PLOS’s free figure tool, NAAS, to help you prepare publication quality figures: https://journals.plos.org/plosone/s/figures#loc-tools-for-figure-preparation.

NAAS will assess whether your figures meet our technical requirements by comparing each figure against our figure specifications.

Attachment Submitted filename: PLOS10825.docx

# Author comment: Author response to Decision Letter 1

**DOI:** 10.1371/journal.pone.0351629.r002

6 May 2026

1.Please ensure that your manuscript meets PLOS ONE's style requirements, including those for file naming. The PLOS ONE style templates can be found at

https://journals.plos.org/plosone/s/file?id=wjVg/PLOSOne_formatting_sample_main_body.pdf and

https://journals.plos.org/plosone/s/file?id=ba62/PLOSOne_formatting_sample_title_authors_affiliations.pdf

Response: The manuscript has been formatted according to the specified requirements.

2. Thank you for stating the following financial disclosure:

“The project was funded by a research grant from the Charles Perkins Centre, The University of Sydney”

Please state what role the funders took in the study. If the funders had no role, please state: "The funders had no role in study design, data collection and analysis, decision to publish, or preparation of the manuscript."

If this statement is not correct you must amend it as needed.

Please include this amended Role of Funder statement in your cover letter; we will change the online submission form on your behalf.

Response: We can confirm that “The funders had no role in study design, data collection and analysis, decision to publish, or preparation of the manuscript."

3. If the reviewer comments include a recommendation to cite specific previously published works, please review and evaluate these publications to determine whether they are relevant and should be cited. There is no requirement to cite these works unless the editor has indicated otherwise.

We have addressed each reviewer comment below:

Reviewer #1

This cross-sectional study by King et al. investigates the relationship between blood pressure and markers of poor oral health among adults aged ≥18 years attending dental clinics in Australia. Although the authors state that this is the first report using Australian data, several concerns should be addressed.

1. In terms of study population and generalizability, the most significant concern is that this study was conducted in a single center and included individuals attending facilities that primarily provide care for low-income populations. While the authors emphasize that this is the first study of its kind in Australia, inclusion of patients from general dental clinics would likely provide more robust and generalizable results.

Response: We agree and this is a limitation of the study. To make this more explicit we have revised the limitations section and included the following wording in the Discussion:

A limitation of this study is that it was conducted at a single site with a convenience sample, this limits the generalisability of the findings to the broader population.

We do already acknowledge that this is an at-risk population and have noted in the Discussion that this study:

provides valuable insights into the health profiles of a population with a high burden of both oral and other chronic diseases, which could be crucial for designing targeted interventions and policies for similar populations in other regions.

2. As noted in both the Methods and Discussion sections, there are concerns regarding the method of blood pressure measurement. However, considering the authors’ point that these measurements reflect real-world practice in dental clinics, it may be more appropriate to revise the definition of “Hypertension” in Table 2 to “Determined hypertension (diagnosed and/or treated) and high measured blood pressure.”

This is because even if the measurement method does not strictly follow guideline-recommended procedures, repeatedly elevated blood pressure readings obtained incidentally may indicate either true hypertension or elevated blood pressure, representing a population at increased risk for future hypertension. (Curr Hypertens Rep. 2012:14:619-25. doi:10.1007/s11906-012-0299-y, BMC Cardiovasc Disord. 2021: 21:523. doi: 10.1186/s12872-021-02334-6.).

Response: Thank you for highlighting this. We have revised the definition of Hypertension in table 2, where we have included a summary of the definition within the table. We have also clarified this in the conclusion.

Discussion: Hypertension as determined in this study was prevalent in this cohort, with lower levels of awareness and treatment among younger compared to older adults.

3. On page 18, regarding hypertension awareness, it would be informative to present the proportion of individuals who are aware of having hypertension or elevated blood pressure and are receiving treatment (or, alternatively, not receiving treatment).

Response: All the people who were treated with anti-hypertensives were aware of their diagnosis. Therefore, the people who are presented in table 2 as being treated are also aware. We have reworded the table to make that clear.

4. In terms of interpretation of the conclusion, the conclusion that “Oral health practitioners could play an important role in primary care by screening younger adults with significant oral disease for undiagnosed or uncontrolled hypertension” is not entirely convincing based on the current data. Further clarification is needed to justify this statement.

Response: we have reworded the abstract conclusion and manuscript conclusion to better reflect the data –

Abstract: In this population with a high burden of oral disease, hypertension was common highlighting the importance of opportunistic blood pressure assessment. Dental settings may offer an additional point of contact for identifying individuals with undiagnosed or uncontrolled hypertension, particularly among younger adults with lower awareness.

Manuscript: Hypertension as determined in this study was prevalent in this cohort, with lower levels of awareness and treatment among younger compared to older adults. These findings underscore the need to improve hypertension awareness and management across adult age groups but particularly in younger adults who may have fewer interactions with medical services. Dental settings may provide a potential venue for opportunistic BP measurement.

Reviewer #2

The article is very well written but needs a stronger statistical corroboration.

1. Methodology of BP recording: Reason for two rather than the recommended three office BP measurements. This is a confounder.

Response: We agree that it would have been more appropriate to record three blood pressure recordings, however due to limitations in time in a real world clinical setting it was only feasible to record two readings. We have acknowledged this as a limitation and added to the discussion –

Discussion:

Another limitation was that due to time constraints only two BP measurements were collected per participant instead of the recommended three, which may have led to an overestimation of true BP levels. However, the study’s BP assessments were conducted in a real-world dental clinic setting …

2. The sample size was meant to detect hypertension. There was no provision for analysis of the correlation between dental problems and HTN and the relevance of the same. The sample size was for a single cohort. But the analysis was for two cohorts.

Response: Thank you, we acknowledge that this study was not powered to detect differences between age cohorts and to identify associations between markers of oral health and hypertension. However, the exploratory analysis did detect significant differences in hypertension prevalence, awareness and treatment between the two age cohorts. We therefore felt it was important to present this. We have now included p-values to support these findings. We have also revised the discussion.

Discussion: Similarly, despite the small sample size, we found that younger individuals were more likely than older individuals to be unaware of their condition, and untreated (29.3% vs 13.2%, 43.9% vs 15.8%, respectively). Hence, screening patients in Australian public dental clinics, especially younger adults is an opportunity to identify and refer those with undiagnosed or uncontrolled hypertension, supporting earlier intervention and better cardiovascular disease prevention. Of those receiving treatment, 43.4% had uncontrolled hypertension, which is higher than the global rate of 38.3%,[31] although similar to the Australian rate of 45.7% [23] and whilst BP control did not differ significantly by age in our study, these findings maybe constrained by the sample size.

We also acknowledge that the finding that there was no association between markers of oral health and HTN may relate to the fact that the study was underpowered and have reworded sections of the discussion as outlined below:

Discussion [First paragraph]: The exploratory analysis did not identify a significant association between markers of poor oral hygiene, significant tooth loss or periodontal treatment needs and hypertension.

Discussion: Exploratory analyses of the relationship between oral health and hypertension found no the association between markers of poor oral hygiene including tooth brushing and interdental cleaning and hypertension. Previous findings from of a meta-analysis that reported a higher risk of hypertension in those with a lower frequency of tooth brushing [10]. However, of the eight studies in this meta-analysis, five were not adjusted for confounders, and the majority were cross- sectional studies. Accordingly, further well designed studies with larger sample sizes are needed to examine these associations.

We have also updated the objective in the abstract and the aims as stated in the introduction to clarify this:

Objective: This study aimed to determine hypertension awareness, treatment and control, among adults attending a public dental clinic, and explore age-related differences between younger (< 65 years) and older adults (≥ 65 years) including associations between markers of oral health and hypertension.

Introduction (Aims): The aim of this study was to determine hypertension awareness, treatment and control, among adults attending a public dental service, and to explore age‑specific patterns and associations with oral health.

3. Overall recruitment rate of 54% will have a selection bias – this may not be a truly representative population. May inflate or deflate the prevalence.

Response: We agree and have included this as a limitation

Additionally, the overall recruitment of 54% may have introduced selection bias which could over- or underestimate the prevalence of hypertension.

We have also updated the results section to clarify that individuals in pain were excluded (this is already noted in figure 1 but we had omitted this from the text in the results):

One of the main reasons for exclusion was the need for an interpreter, other reasons included lack of participant time or interest in participation or if the individual was in pain (Figure 1).

4. When two groups are mentioned in Table 1 – the p value for the differences are not mentioned. This is important to know the baseline difference between the groups. Table 2 also needs similar p values. The study was not powered for these analyses, but these are done

Response: The p-values have been added to table 1 and table 2 and additional text has been included in the statistical analysis section:

Differences in proportions between groups were calculated using a chi-square test.

5. Hypertension prevalence increases with age, and this is quite striking. Needs mention

Response: Thank you for drawing our attention to this, we have already mentioned this finding in the results and have revised the first paragraph of the discussion to highlight the key findings:

Discussion: A higher proportion of older (74.1%) compared to younger adults (28.1%) in this population had hypertension and while overall rates of awareness and treatment were high, younger adults were less likely than older adults to be aware and treated for hypertension (56.1% vs 84.2%, 30.0% vs 47.4%, respectively). These findings suggest that within public dental services there are opportunities to address broader cardiovascular disease prevention goals, especially among younger adults.

6. Para 1 of discussion – two inferences – the overall treatment and awareness percentages? without difference in age and the poor treatment and control among younger individuals needs statistical correlation

Response: We have included the p-values in Table 2 which demonstrate that the proportion of adults with hypertension awareness and treatment is significantly lower in younger compared to older adults.

We analysed the untreated numbers as well (see below), however did not include this data in the table as we did not feel they contributed any further information. The presentation of data in Table 2 is aligned with data presentation in the May Measurement Month campaign ref: Carnagarin et al 2021 ( https://doi.org/10.1093/eurheartj/suab016).

Additionally, we have reworded the first paragraph of the discussion in response to comment #5 and feel that this has now been addressed.

Hypertension (Untreated)

[Number (%) of patients aware and untreated among those with hypertension, missing=1] 9/156

(5.8%)

(CI: 3.1%, 10.6%)

6/23

(26.1%)

(CI: 112.5%, 46.5%) 3/96

(3.1%)

(CI: 1.1%, 8.8%) 0.002

7. The dental correlation is underpowered.

Response: We have acknowledged this and revised several sections of the manuscript including the Abstract, Introduction and Discussion – please see response to comment #2 for details.

8. Limitation – underpowered to correlate the oral pathology with hypertension. 54% recruitment must be justified. Confounders need clear mention - the role of pain and its impact on the control, two measurements rather than three that can falsely elevate BP.

Response:

- We have addressed the issue of the study being underpowered in our response to comment #2 .

- We have addressed the 54% response rate (see response to comment #3)

- We have clarified that individuals in pain were excluded (figure 1 and response to comment #3).

- We have addressed the two blood pressure measurements and acknowledged this as a limitation (response to comment # 1)

10. The statement that screening among younger is more beneficial needs stress

Response: Thank you for this comment, we have revised the first paragraph of the discussion to highlight this (see response to comment #5), we have also revised the conclusion to highlight this point:

Hypertension as determined in this study was prevalent in this cohort, with lower levels of awareness and treatment among younger compared to older adults. These findings underscore the need to improve hypertension awareness and management across adult age groups but particularly in younger adults who may have fewer interactions with medical services. Dental settings may be a potential venue for opportunistic BP measurement.

Attachment Submitted filename: Response to reviewers.docx

# Peer review: Decision Letter 1

**DOI:** 10.1371/journal.pone.0351629.r003

**Authors:** Masaki Mogi

20 May 2026

<div>PONE-D-26-10825R1

Screening for hypertension in a public dental clinic: A single-centre cross-sectional study in Australia

PLOS One

Dear Dr. King,

Thank you for submitting your manuscript to PLOS ONE. After careful consideration, we feel that it has merit but does not fully meet PLOS ONE’s publication criteria as it currently stands. Therefore, we invite you to submit a revised version of the manuscript that addresses the points raised during the review process.

==============================

Monor revisions are necessary in the prsent form.

==============================

Please submit your revised manuscript by Jul 04 2026 11:59PM. If you will need more time than this to complete your revisions, please reply to this message or contact the journal office at plosone@plos.org. When you're ready to submit your revision, log on to https://www.editorialmanager.com/pone/ and select the 'Submissions Needing Revision' folder to locate your manuscript file.

Please include the following items when submitting your revised manuscript:

- A letter that responds to each point raised by the academic editor and reviewer(s). You should upload this letter as a separate file labeled 'Response to Reviewers'.
- A marked-up copy of your manuscript that highlights changes made to the original version. You should upload this as a separate file labeled 'Revised Manuscript with Track Changes'.
- An unmarked version of your revised paper without tracked changes. You should upload this as a separate file labeled 'Manuscript'.

If you would like to make changes to your financial disclosure, please include your updated statement in your cover letter. Guidelines for resubmitting your figure files are available below the reviewer comments at the end of this letter.

If applicable, we recommend that you deposit your laboratory protocols in protocols.io to enhance the reproducibility of your results. Protocols.io assigns your protocol its own identifier (DOI) so that it can be cited independently in the future. For instructions see: https://journals.plos.org/plosone/s/submission-guidelines#loc-laboratory-protocols. Additionally, PLOS ONE offers an option for publishing peer-reviewed Lab Protocol articles, which describe protocols hosted on protocols.io. Read more information on sharing protocols at https://plos.org/protocols?utm_medium=editorial-email&utm_source=authorletters&utm_campaign=protocols.

As the corresponding author, your ORCID iD is verified in the submission system and will appear in the published article. PLOS supports the use of ORCID, and we encourage all coauthors to register for an ORCID iD and use it as well. Please encourage your coauthors to verify their ORCID iD within the submission system before final acceptance, as unverified ORCID iDs will not appear in the published article. *Only*   the individual author can complete the verification step; PLOS staff *cannot*   verify ORCID iDs on behalf of authors.

We look forward to receiving your revised manuscript.

Kind regards,

Masaki Mogi

Academic Editor

PLOS One

Journal Requirements:

If the reviewer comments include a recommendation to cite specific previously published works, please review and evaluate these publications to determine whether they are relevant and should be cited. There is no requirement to cite these works unless the editor has indicated otherwise.

Please review your reference list to ensure that it is complete and correct. If you have cited papers that have been retracted, please include the rationale for doing so in the manuscript text, or remove these references and replace them with relevant current references. Any changes to the reference list should be mentioned in the rebuttal letter that accompanies your revised manuscript. If you need to cite a retracted article, indicate the article’s retracted status in the References list and also include a citation and full reference for the retraction notice.

[Note: HTML markup is below. Please do not edit.]

Reviewers' comments:

Reviewer's Responses to Questions

**Comments to the Author**

1. If the authors have adequately addressed your comments raised in a previous round of review and you feel that this manuscript is now acceptable for publication, you may indicate that here to bypass the “Comments to the Author” section, enter your conflict of interest statement in the “Confidential to Editor” section, and submit your "Accept" recommendation.

Reviewer #1: All comments have been addressed

Reviewer #2: All comments have been addressed

**********

2. Is the manuscript technically sound, and do the data support the conclusions?

The manuscript must describe a technically sound piece of scientific research with data that supports the conclusions. Experiments must have been conducted rigorously, with appropriate controls, replication, and sample sizes. The conclusions must be drawn appropriately based on the data presented.

Reviewer #1: (No Response)

Reviewer #2: Partly

**********

3. Has the statistical analysis been performed appropriately and rigorously?

Reviewer #1: (No Response)

Reviewer #2: Yes

**********

4. Have the authors made all data underlying the findings in their manuscript fully available?

The PLOS Data policy requires authors to make all data underlying the findings described in their manuscript fully available without restriction, with rare exception (please refer to the Data Availability Statement in the manuscript PDF file). The data should be provided as part of the manuscript or its supporting information, or deposited to a public repository. For example, in addition to summary statistics, the data points behind means, medians and variance measures should be available. If there are restrictions on publicly sharing data—e.g. participant privacy or use of data from a third party—those must be specified.

Reviewer #1: (No Response)

Reviewer #2: Yes

**********

5. Is the manuscript presented in an intelligible fashion and written in standard English?

PLOS ONE does not copyedit accepted manuscripts, so the language in submitted articles must be clear, correct, and unambiguous. Any typographical or grammatical errors should be corrected at revision, so please note any specific errors here.

Reviewer #1: (No Response)

Reviewer #2: Yes

**********

6. Review Comments to the Author

Please use the space provided to explain your answers to the questions above. You may also include additional comments for the author, including concerns about dual publication, research ethics, or publication ethics. (Please upload your review as an attachment if it exceeds 20,000 characters)

Reviewer #1: Although there are several limitations, the authors have responded appropriately to the reviewers’ questions.

Reviewer #2: Overall revised version is done well.

Minor corections -

1. the prevalence of HTN in Australia - 22 to 34% in different portions - the statistical and introduction. may be better to give a range - which is quite acceptable

2. the total number is 302 but the < 65 and > 65 years category adds upto 301 - if this is corrected then most of the numbers have to be carefully reworked. my not change the conclusion.

3. In the conclusion, it might be good to add that the prevalence of HTN in patients attending the dental clinic is higher and provides a very good opportunity to scren a population with a higher prevalence than the population.This will emphasize the importance of the study .

**********

7. PLOS authors have the option to publish the peer review history of their article (what does this mean?). If published, this will include your full peer review and any attached files.

If you choose “no”, your identity will remain anonymous but your review may still be made public.

**Do you want your identity to be public for this peer review?**   For information about this choice, including consent withdrawal, please see our Privacy Policy.

Reviewer #1: No

Reviewer #2: **Yes:**  Prabhakar Dorairaj

**********

[NOTE: If reviewer comments were submitted as an attachment file, they will be attached to this email and accessible via the submission site. Please log into your account, locate the manuscript record, and check for the action link "View Attachments". If this link does not appear, there are no attachment files.]

To ensure your figures meet our technical requirements, please review our figure guidelines: https://journals.plos.org/plosone/s/figures

You may also use PLOS’s free figure tool, NAAS, to help you prepare publication quality figures: https://journals.plos.org/plosone/s/figures#loc-tools-for-figure-preparation.

NAAS will assess whether your figures meet our technical requirements by comparing each figure against our figure specifications.

# Author comment: Author response to Decision Letter 2

**DOI:** 10.1371/journal.pone.0351629.r004

28 May 2026

1. the prevalence of HTN in Australia - 22 to 34% in different portions - the statistical and introduction. may be better to give a range - which is quite acceptable

Response: Thank you we have provided a range of 22-34% in the introduction.

2. the total number is 302 but the < 65 and > 65 years category adds upto 301 - if this is corrected then most of the numbers have to be carefully reworked. my not change the conclusion.

Response: The number of participants in the age categories does not add up to 302 because one participant did not provide their age, we noted missingness in Table 2 but did not note missingness in Table 1. We have now included missingness for all variables in Table 1 to ensure greater clarity.

3. In the conclusion, it might be good to add that the prevalence of HTN in patients attending the dental clinic is higher and provides a very good opportunity to scren a population with a higher prevalence than the population. This will emphasize the importance of the study.

Response: We have revised the conclusion to make this point more clearly –

The proportion with hypertension as determined in this study was higher than the national average, with lower levels of awareness and treatment among younger compared to older adults. These findings underscore the need to improve hypertension awareness and management across adult age groups but particularly in younger adults who may have fewer interactions with medical services. Dental settings may provide a potential venue for opportunistic BP measurement in a population with a higher prevalence of hypertension.

Attachment Submitted filename: Response to reviewers 2.docx

# Peer review: Decision Letter 2

**DOI:** 10.1371/journal.pone.0351629.r005

**Authors:** Masaki Mogi

1 Jun 2026

Screening for hypertension in a public dental clinic: A single-centre cross-sectional study in Australia

PONE-D-26-10825R2

Dear Dr. King,

We’re pleased to inform you that your manuscript has been judged scientifically suitable for publication and will be formally accepted for publication once it meets all outstanding technical requirements.

Within one week, you’ll receive an e-mail detailing the required amendments. When these have been addressed, you’ll receive a formal acceptance letter and your manuscript will be scheduled for publication.

An invoice will be generated when your article is formally accepted. Please note, if your institution has a publishing partnership with PLOS and your article meets the relevant criteria, all or part of your publication costs will be covered. Please make sure your user information is up-to-date by logging into Editorial Manager at Editorial Manager® and clicking the ‘Update My Information' link at the top of the page. For questions related to billing, please contact billing support.

If your institution or institutions have a press office, please notify them about your upcoming paper to help maximize its impact. If they’ll be preparing press materials, please inform our press team as soon as possible -- no later than 48 hours after receiving the formal acceptance. Your manuscript will remain under strict press embargo until 2 pm Eastern Time on the date of publication. For more information, please contact onepress@plos.org.

Kind regards,

Masaki Mogi

Academic Editor

PLOS One

Additional Editor Comments (optional):

Reviewers' comments:

Reviewer's Responses to Questions

**Comments to the Author**

1. If the authors have adequately addressed your comments raised in a previous round of review and you feel that this manuscript is now acceptable for publication, you may indicate that here to bypass the “Comments to the Author” section, enter your conflict of interest statement in the “Confidential to Editor” section, and submit your "Accept" recommendation.

Reviewer #2: All comments have been addressed

**********

2. Is the manuscript technically sound, and do the data support the conclusions?

The manuscript must describe a technically sound piece of scientific research with data that supports the conclusions. Experiments must have been conducted rigorously, with appropriate controls, replication, and sample sizes. The conclusions must be drawn appropriately based on the data presented.

Reviewer #2: Yes

**********

3. Has the statistical analysis been performed appropriately and rigorously?

Reviewer #2: Yes

**********

4. Have the authors made all data underlying the findings in their manuscript fully available?

The PLOS Data policy requires authors to make all data underlying the findings described in their manuscript fully available without restriction, with rare exception (please refer to the Data Availability Statement in the manuscript PDF file). The data should be provided as part of the manuscript or its supporting information, or deposited to a public repository. For example, in addition to summary statistics, the data points behind means, medians and variance measures should be available. If there are restrictions on publicly sharing data—e.g. participant privacy or use of data from a third party—those must be specified.

Reviewer #2: Yes

**********

5. Is the manuscript presented in an intelligible fashion and written in standard English?

PLOS ONE does not copyedit accepted manuscripts, so the language in submitted articles must be clear, correct, and unambiguous. Any typographical or grammatical errors should be corrected at revision, so please note any specific errors here.

Reviewer #2: Yes

**********

6. Review Comments to the Author

Please use the space provided to explain your answers to the questions above. You may also include additional comments for the author, including concerns about dual publication, research ethics, or publication ethics. (Please upload your review as an attachment if it exceeds 20,000 characters)

Reviewer #2: all comments addressed.

the article may be published

the conclusion now highlights the importance of this research

**********

7. PLOS authors have the option to publish the peer review history of their article (what does this mean?). If published, this will include your full peer review and any attached files.

If you choose “no”, your identity will remain anonymous but your review may still be made public.

**Do you want your identity to be public for this peer review?**   For information about this choice, including consent withdrawal, please see our Privacy Policy.

Reviewer #2: No

**********

# Editor report: Acceptance letter

**DOI:** 10.1371/journal.pone.0351629.r006

**Authors:** Masaki Mogi

PONE-D-26-10825R2

PLOS One

Dear Dr. King,

I'm pleased to inform you that your manuscript has been deemed suitable for publication in PLOS One. Congratulations! Your manuscript is now being handed over to our production team.

At this stage, our production department will prepare your paper for publication. This includes ensuring the following:

* All references, tables, and figures are properly cited

* All relevant supporting information is included in the manuscript submission,

* There are no issues that prevent the paper from being properly typeset

You will receive further instructions from the production team, including instructions on how to review your proof when it is ready. Please keep in mind that we are working through a large volume of accepted articles, so please give us a few days to review your paper and let you know the next and final steps.

Lastly, if your institution or institutions have a press office, please let them know about your upcoming paper now to help maximize its impact. If they'll be preparing press materials, please inform our press team within the next 48 hours. Your manuscript will remain under strict press embargo until 2 pm Eastern Time on the date of publication. For more information, please contact onepress@plos.org.

You will receive an invoice from PLOS for your publication fee after your manuscript has reached the completed accept phase. If you receive an email requesting payment before acceptance or for any other service, this may be a phishing scheme. Learn how to identify phishing emails and protect your accounts at https://explore.plos.org/phishing.

If we can help with anything else, please email us at customercare@plos.org.

Thank you for submitting your work to PLOS ONE and supporting open access.

Kind regards,

PLOS ONE Editorial Office Staff

on behalf of

Dr. Masaki Mogi

Academic Editor

PLOS One
